# Supplementary material for: Tracheal branching in ants is area-decreasing, violating a central assumption of network transport models
Source: PLoS Comput Biol. 2020 Apr 30;16(4):e1007853. doi: 10.1371/journal.pcbi.1007853 (PMC7241831; doi:10.1371/journal.pcbi.1007853)
Supplement: S1 Text — Studien über Tracheenrespiration II1). Über Gasdiffusion in den Tracheen. Pflügers Archiv 179: 95–112. (PDF) [file pcbi.1007853.s001.pdf]

Supporting Information S1

Tracheal branching in ants is area-decreasing, violating a central assumption of network transport models

Ian J. Aitkenhead[1], Grant A. Duffy[1], Citsabehsan Devendran[2], Michael R. Kearney[3], Adrian Neild[2] and Steven L. Chown[1,*]

1 School of Biological Sciences, Monash University, Victoria 3800, Australia, **2** Department of Mechanical and Aerospace Engineering, Monash University, Victoria 3800, Australia, **3** School of BioSciences, University of Melbourne, Victoria 3010, Australia

*steven.chown@monash.edu

Translation of pages 98-100 of

Krogh, A. 1920. Studien über Tracheenrespiration II[1]). Über Gasdiffusion in den Tracheen. Pflügers Archiv 179: 95-112.

Krogh, A. 1920. Studies on Tracheal Respiration II[1]). Concerning Gas Diffusion in the Tracheae. Pflügers Archiv 179: 95-112.

The text below is a translation of pages 98 to 100 in Krogh's text. We have not sought to change the units he provides or interpret his statements, but simply to render an English version of the German original (the original text of which is reproduced below the English Version). The only change we have made is that the boldfaced type has been added here for emphasis and is not so in the original.

The first measurements were made on alcohol specimens from the European Myriapod Scutigera, whose tracheal system is particularly simply built. Seven unpaired stigmas are found in the middle of the dorsal line in this animal. Every stigma leads to an air space, from which lead after Haase[1]) about 600 proportionally short tracheae, which branch a few times dichotomously. I have the average length of trachea as 0,55 mm (5,5 x 10^{-2} cm) and the average diameter of the single trachea trunks as 0.015 cm. The cross-sectional area is then 1,7x 10^{-6} qcm. I was convinced that the sum of the cross section of the branches is approximately the same as that of the trunk. The probable oxygen consumption can be up to about 350 cc per kilogram and hour, which, if the weight is set at 0.3 g, gives a consumption of 3 x 10^{-5} cc per animal and per second. One has then

$$3 * 10^{-5} = 0{,}18(p - p1)\frac{7 * 600 * 1{,}7 * 10^{-6}}{5.5 * \ 10^{-2}}$$

and hence $p - p_1$ = 0.0013 or 0.13% of an atmosphere.

The calculation shows that an oxygen percentage of 20.8 in the inner ends of the tracheal tubes compared with 20.93 in the free atmosphere is sufficient for the normal needs of the animal allowing oxygen diffusion through the trachea. Breathing movements are therefore completely superfluous.

A similar calculation was then made for mealworms (larvae of Tenebrio). The gas exchange has been determined several times here and for an animal of 0.3 g weight can be considered 3x10^{-5} ccm per second. A simple dissection shows that the tracheal system consists mainly of small groups of on average four tracheal tubes emanating from the stigmata. The total number of primary tracheae is about 80, and the diameter varies between 50 µ and 70 µ (average 60 µ). The branching is very abundant, but could not be followed in detail. **I have arbitrarily assumed that the total cross-section at the branch does not change**, and also estimated the average length as 3 mm. I land thus

$$3 * 10^{-5} = 0{,}18(p - p1)\frac{80 * \pi * 30^2 * 10^{-8}}{0.3}$$

and hence $p - p_1$ = 0.022 or 2.2% of an atmosphere. Here as well diffusion will suffice completely for the air renewal in the trachea, and breathing movements are superfluous

It is obvious that in the cited cases and especially for Tenebrio the numerical results can claim only a very limited accuracy. So I've been trying to find a method that would allow more accurate measurements. What should be achieved, is a concise preparation of the entire tracheal system, combined with a staining that makes even the finest tracheal branches visible and allows measurements of their lumens. I have made such very useful preparations by injection of the whole tracheal system with solidifying fat, possibly intensively colored by Alkanna (or Sudan) and subsequent digestion of the tissues obtained by means of pepsin hydrochloric acid. The details of the method have been published elsewhere. Fig. 2 shows such a preparation of a Cossus larva.

I have a larger number of such prepared specimens and some of them are used for tracheal measurements. An examination of the pictured specimen shows that a complete measurement of all tracheal branches would require a great deal of work. So I've simplified the task for myself. First, by inspection of the measuring preparations I have found that of the different spiracles the outgoing trachea among themselves were not strikingly different and therefore some could be selected as typical for the measurement. Of these, the tracheal stems were then removed one at a time by means of fine scissors, spread under the microscope and measured.

I began to investigate the extent of a change of the total transverse section with the junction. **It has been found that a stem cross section is usually a little larger than the sum of the branch cross sections.** I present the following measurements. The numbers are micrometer readings with Obj. A 0 (Zeiss Binocular Microscope). I simply compare the square of the stem diameter (D^2) with the square sum of the branch diameter Σd^2.

Table 1

Stem D	Branches d	D^2	Σd^2
4	2,5<br>2<br>1,7	16	13,2
5	4<br>3<br>2	25	29
6	3 x 2<br>3 x 3	36	35
9	2 x 2<br>3 x 2,5<br>3<br>2 x 4	81	68
11	2 x 3,5<br>2 x 4,5<br>2 x 5	121	115
		279	260

The difference between D^2 and Σ d^2 is quite insignificant (7%), and one is thus entitled to measure the cross-section of each tracheal stem only in one level. In the main

measurements I have, as far as possible, the cross sections not of the stems, but those measured after the first branching. At each tracheal branch the average length of the branches, calculated from the spiracle has also been determined. These measurements were done by means of the lens F55. The micrometer values for F55 are 1 graduation = 0,082 mm, for a_0 1 graduation = 0,0465 mm.

I took the following measurements on a larva of Cossus ligniperda of 3,4 g liveweight. The spiracles connecting longitudinal stems are not included.

Translation by S.L. Chown

Länge (l) aller Tracheenzweige, von den Spirakeln aus gerechnet, zu bestimmen. Diese Längen sind sehr verschieden, und die allerletzten Verzweigungen können gar nicht gesehen oder gemessen werden. Es gilt ferner den gesamten Querschnitt aller Tracheen (a) in den verschiedenen Abständen von den Spirakeln zu messen. Auch diese Aufgabe kann natürlich nur approximativ gelöst werden.

Die ersten Messungen wurden an Spiritusexemplaren von der südeuropäischen Myriapode Scutigera, deren Tracheensystem besonders einfach gebaut ist, angestellt. An diesem Tiere finden sich in der Mitte der Rückenlinie sieben ungepaarte Stigmen. Jedes Stigma führt in eine Lufthöhle, in welcher nach Haase[1]) etwa 600 verhältnismässig kurze, sich einige Male dichotomisch verzweigende Tracheen einmünden. Ich habe die durchschnittliche Länge der Tracheen zu 0,55 mm ($5{,}5 \times 10^{-2}$ cm) gemessen und der durchschnittliche Diameter der einzelnen Tracheenstämme zu 0,015 mm. Das Querschnittsareal ist dann $1{,}7 \times 10^{-6}$ qcm. Ich habe mich davon überzeugt, dass die Summe der Ästchenquerschnitte ungefähr denen des Stammes gleich ist. Der wahrscheinliche Sauerstoffverbrauch lässt sich zu ungefähr 350 ccm pro Kilogramm und Stunde abschätzen, welcher, wenn das Gewicht zu 0,3 g angesetzt wird, einem Verbrauch von 3×10^{-5} ccm pro Tier und pro Sekunde gibt. Man hat dann

$$3 \times 10^{-5} = 0{,}18\,(p-p_1)\,\frac{7 \times 600 \times 1{,}7 \times 10^{-6}}{5{,}5 \times 10^{-2}},$$

und folglich $p-p_1 = 0{,}0013$ oder 0,13 % einer Atmosphäre.

Die Berechnung zeigt, dass ein Sauerstoffprozent von 20,8 in den inneren Enden der Tracheenröhren im Vergleich mit 20,93 in der freien Atmosphäre eine für die normalen Bedürfnisse des Tieres genügende Sauerstoffdiffusion durch die Tracheen erlauben wird. Atembewegungen sind somit ganz überflüssig.

Eine ähnliche Berechnung wurde dann für Mehlwürmer (Larven von Tenebrio) angestellt. Der Gaswechsel ist hier mehrmals bestimmt worden und kann für ein Tier von 0,3 g Gewicht auf 3×10^{-5} ccm pro Sekunde angesetzt werden. Eine einfache Dissektion zeigt, dass das Tracheensystem hauptsächlich aus kleinen Gruppen von durchschnittlich vier Tracheenröhren, die von den Stigmen ausgehen, besteht. Die gesamte Anzahl der Primärtracheen ist ungefähr 80, und der Durchmesser variiert zwischen 50 und 70 μ (Mittel 60 μ). Die Verzweigung ist sehr reichlich, konnte aber nicht im Detail verfolgt werden. Ich habe willkürlich angenommen, dass sich der Gesamt-

1) Haase hat sehr schöne und genaue Abbildungen der Tracheenlungen gegeben. Leider fehlen aber, wie es öfters in zoologischen Publikationen der Fall ist, Grössenangaben gänzlich, so dass die Abbildungen sich nicht für Ausmessungen verwerten lassen.

querschnitt bei der Verzweigung nicht ändert, und ferner die mittlere Länge auf 3 mm geschätzt. Ich fand somit

$$3 \times 10^{-5} = 0{,}18\,(p-p_1)\,\frac{80 \times \pi \times 30^2 \times 10^{-8}}{0{,}3},$$

und folglich $p-p_1 = 0{,}022$ oder 2,2% einer Atmosphäre. Also auch hier wird Diffusion für die Lufterneuerung in den Tracheen vollständig genügen, und Atembewegungen sind überflüssig

Es liegt auf der Hand, dass in den genannten Fällen und besonders für Tenebrio die zahlenmässigen Resultate nur eine sehr beschränkte Genauigkeit beanspruchen können. Ich habe mich daher bemüht, eine Methode ausfindig zu machen, die genauere Messungen gestatten würde. Was erreicht werden sollte, war eine übersichtliche Präparation des gesamten Tracheensystems, mit einer Färbung kombiniert, die auch die feinsten Tracheenästchen deutlich sichtbar macht und Messungen ihrer Lumina gestattete. Solche sehr brauchbare Präparate habe ich durch Injektion des ganzen Tracheensystems mit erstarrendem, durch Alkanna (oder Sudan) möglichst intensiv gefärbtem Fett und nachherige Verdauung der Gewebe mittelst Pepsinsalzsäure gewonnen. Die Details der Methodik habe ich anderswo beschrieben[1]). Abb. 2 zeigt ein solches Präparat von einer Cossuslarve.

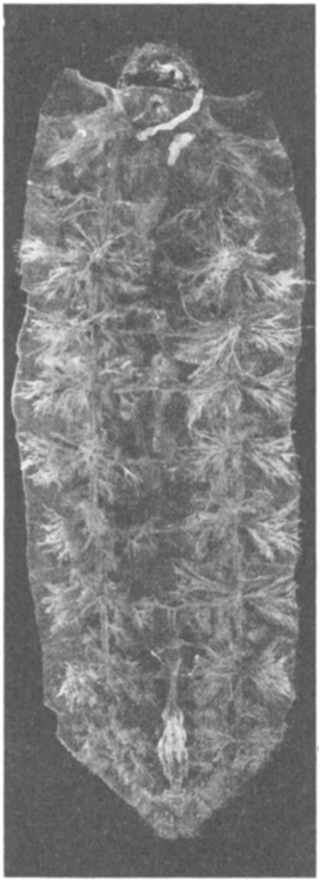

Abb. 2.

Ich habe eine grössere Anzahl solcher Präparate angefertigt[2]) und einige derselben für Tracheenmessungen verwertet. Eine Betrachtung des abgebildeten Präparats zeigt, dass eine vollständige Ausmessung aller Tracheenzweige einen überaus grossen Arbeitsaufwand beanspruchen würde. Ich habe mir daher die Aufgabe vereinfacht. Zunächst wurde durch Inspektion des zu

1) Krogh, Injection preparation of the tracheal system of insects Vid. Medd. Dansk naturh. Forening 68. 1917.

2) Die Herren Entomologen Lehrer J. Kryger und Dr. phil. J. C. Nielsen haben mich auf freundlichste Weise mit Material unterstützt. Ich spreche ihnen dafür meinen besten Dank aus.

messenden Präparates festgestellt, dass die von den verschiedenen Spirakeln ausgehenden Tracheensträucher untereinander nicht auffallend verschieden waren und daher einige als typisch für die Messung ausgewählt werden konnten. Von diesen wurden dann die Tracheenstämme einzeln mittelst feiner Schere entnommen, unter dem Mikroskop ausgebreitet und gemessen.

Ich fing damit an zu untersuchen, inwieweit sich eine Änderung des Gesamtquerschnittes mit der Verzweigung einstellt. Es wurde festgestellt, dass ein Stammquerschnitt gewöhnlich ein wenig grösser ist als die Summe der Ästenquerschnitte. Ich gebe zum Beleg die folgenden Messungen. Die Zahlen sind Mikrometerablesungen mit Obj. a_0 (Zeiss-Binokularmikroskop). Ich vergleiche einfach das Quadrat des Stammdurchmessers (D^2) mit der Quadratsumme der Ästendurchmesser $\Sigma\, d^2$.

Tabelle I.

Stamm D	Ästchen d	D^2	Σd^2
4	2,5<br>2<br>1,7	16	13,2
5	4<br>3<br>2	25	29
6	3 × 2<br>3 × 3	36	35
9	2 × 2<br>3 × 2,5<br>3<br>2 × 4	81	68
11	2 × 3,5<br>2 × 4,5<br>2 × 5	121	115
		279	260

Der Unterschied zwischen D^2 und $\Sigma\, d^2$ ist recht unbedeutend (7 %), und man ist somit berechtigt, an jedem Tracheenstamm die Querschnittsmessung nur in einem Niveau durchzuführen. In den Hauptmessungen habe ich, soweit möglich, die Querschnitte nicht an den Stämmen, sondern nach der ersten Verzweigung gemessen. An jedem Tracheenbaum ist ferner die durchschnittliche Länge der Zweige, vom Spirakel aus gerechnet, bestimmt worden. Diese Messungen geschahen mittelst des Objektivs F_{55}. Die Mikrometerwerte sind für F_{55} 1 Einteilung = 0,082 mm, für a_0 1 Einteilung = 0,0465 mm.

An einer Larve von Cossus ligniperda von 3,4 g Lebendgewicht habe ich die folgenden Messungen angestellt. Die die Spirakeln verbindenden Längsstämme sind nicht mitgerechnet.
