# Supplementary material for: Tracheal branching in ants is area-decreasing, violating a central assumption of network transport models
Source: PLoS Comput Biol. 2020 Apr 30;16(4):e1007853. doi: 10.1371/journal.pcbi.1007853 (PMC7241831; doi:10.1371/journal.pcbi.1007853)
Supplement: S2 Text — (PDF) [file pcbi.1007853.s002.pdf]

Supporting Information S4

Tracheal branching in ants is area-decreasing, violating a central assumption of network transport models

Ian J. Aitkenhead[1], Grant A. Duffy[1], Citsabehsan Devendran[2], Michael R. Kearney[3], Adrian Neild[2] and Steven L. Chown[1,*]

1 School of Biological Sciences, Monash University, Victoria 3800, Australia, **2** Department of Mechanical and Aerospace Engineering, Monash University, Victoria 3800, Australia, **3** School of BioSciences, The University of Melbourne, Victoria 3010, Australia

Correspondence: steven.chown@monash.edu

Analytical Derivation of Diffusion

Accommodating for CO_2 influx into the tracheal network from the tissue

We begin with

$$f_{x+\Delta x} = f_{\Delta x} + A_s\,\varphi \quad (1)$$

where, φ is the CO_2 flux per unit area into the tracheal network from the tissue, x is the distance from the start of the branch level and Δx is an infinitesimal length. The surface area is given as, $A_s = 2\pi rx$ where, r is the radius and flux per infinitesimal length is given by, $f_{\Delta x} = 2\pi r\Delta x\varphi$.

Also, we know from Fick's law

$$f_{x+\Delta x} = \frac{KA}{\Delta x}\Delta pp_{\Delta x} \quad (2a)$$

Rearranged in terms of $\Delta pp_{\Delta x}$,

$$\Delta pp_{\Delta x} = f_{x+\Delta x}\frac{\Delta x}{\mathrm{KA}} \quad (2b)$$

where, the cross-sectional area, $A=\pi r^2$ and K is the Krogh's constant [1].

Substituting Equation 1 into Equation 2b, we obtain

$$\Delta pp_{\Delta x} = \frac{2\varphi}{Kr}[\Delta x^2 + x\Delta x] \quad (3)$$

Integrate Equation 3 over the length of the branch level, L and assuming Δx^2 is negligible, we arrive at

$$\Delta pp_L = \int_0^L \frac{2\varphi}{Kr}x dx = \left[\frac{x^2\varphi}{Kr}\right]_0^L = \frac{L^2\varphi}{Kr} \quad (4)$$

We let,

$$\varphi = \frac{F}{2\pi rL} \quad (5)$$

Substituting Equation 5 in Equation 4, we obtain

$$\Delta pp_{L_n} = \frac{F_n}{2KA}L_n \quad (6)$$

where, n is the branch level number and F_n is the total influx of CO_2 into the tracheal network from the tissue at each branch of corresponding level.

Similarly, for following branches with influx of CO_2 from the tissue, accommodating for the influx from the previous branch (i.e the deeper branch), $F_{n+1,T}$, we get

$$\Delta pp_{L_n} = \frac{F_n}{2KA} L_n + \frac{F_{n+1,T}}{KA} L_n \qquad (7)$$

We carry on with the remaining branch levels (without CO_2 influx from the tissue; i.e. Level 1 and 2). Using Fick's law as written in Equation 2, we obtain

$$\Delta pp_{L_n} = \frac{F_{n+1,T}}{KA} L_n \qquad (8)$$

Flux Calculations

To obtain the flux from level 5 to level 4, we use Equation 6,

$$pp_5 - pp_4 = \frac{F_5 L_5}{2KA_5} \qquad (9)$$

Similarly, for level 4 to level 3, we use Equation 7,

$$pp_4 - pp_3 = \frac{F_4 L_4}{2KA_4} + \frac{2F_5 L_4}{KA_4} \qquad (10)$$

where, $F_{5,T} = 2F_5$ (assuming steady state). Total outward flux, $F_{5,T}$ is the sum of the two child branches, F_5.

Rearranging Equation 9 in terms of pp_4 and substituting into Equation 10 we get,

$$pp_5 - pp_3 = \frac{L_4}{KA_4}\left[\frac{F_4}{2} + 2F_5\right] \qquad (11)$$

We repeat the process above to calculate the flux between level 3 and level 2 using Equation 7 and level 2 to level 1 using Equation 8, we obtain

$$pp_5 - pp_1 = 2F_{3,T}\frac{L_2}{2KA_2} + \frac{L_3}{KA_3}\left[\frac{F_3}{2} + 2F_{4,T}\right] + \frac{L_4}{KA_4}\left[\frac{F_4}{2} + 2F_5\right] \qquad (12)$$

where, $F_{2,T} = 2F_{3,T}$ (assuming steady state).

Finally, for level 1 and atmosphere using Equation 8, assuming steady state, we arrive at

$$pp_5 - pp_{atm} = 4F_{3,T}\frac{L_1}{2KA_1} + 2F_{3,T}\frac{L_2}{2KA_2} + \frac{L_3}{KA_3}\left[\frac{F_3}{2} + 2F_{4,T}\right] + \frac{L_4}{KA_4}\left[\frac{F_4}{2} + 2F_5\right] \qquad (13)$$

where, $F_{1,T} = 2F_{2,T} = 4F_{3,T}$ (assuming steady state).

Now to obtain the arbitrary value of F_5, we assume a constant flux per unit area, φ

$$\varphi_5 = \varphi_4 = \varphi_3 \qquad (14a)$$

therefore,

$$\frac{F_5}{r_5 L_5} = \frac{F_4}{r_4 L_4} = \frac{F_3}{r_3 L_3} \qquad (14b)$$

Substituting Equation 14b into Equation 13, along with $F_{3,T} = 4F_5 + 2F_4 + F_3$ and $F_{4,T} = 2F_5 + F_4$ we obtain,

$$pp_5 - pp_{atm} = \frac{2}{K}\left[4F_5 + 2F_5\frac{r_4L_4}{r_5L_5} + F_5\frac{r_3L_3}{r_5L_5}\right]\left[\frac{2L_1}{A_1} + \frac{L_2}{A_2}\right] + \frac{L_3}{KA_3}\left[F_5\frac{r_3L_3}{2r_5L_5} + 2\left(2F_5 + F_5\frac{r_4L_4}{r_5L_5}\right)\right] + \frac{L_4}{KA_4}\left[2F_5 + F_5\frac{r_4L_4}{2r_5L_5}\right] \quad (15)$$

Rearranging Equation 15 to find F_5, we arrive at

$$F_5 = \frac{pp_5 - pp_{atm}}{T_1 + T_2 + T_3} \quad (16)$$

where,

$$T_1 = \frac{2}{K}\left[4 + 2\frac{r_4L_4}{r_5L_5} + \frac{r_3L_3}{r_5L_5}\right]\left[\frac{2L_1}{A_1} + \frac{L_2}{A_2}\right] \quad (17a)$$

$$T_2 = \frac{L_3}{KA_3}\left[\frac{r_3L_3}{2r_5L_5} + 4 + 2\frac{r_4L_4}{r_5L_5}\right] \quad (17b)$$

$$T_3 = \frac{L_4}{KA_4}\left[2 + \frac{r_4L_4}{2r_5L_5}\right] \quad (18c)$$

Assuming, $pp_5 - pp_{atm} = \Delta pp_{CO_2} = 6$ kPa, the Krogh's constant, K = 1.428104 × 10^{-10} m^2 s^{-1} Pa^{-1} [1] and the geometrical lengths and radii are known, we can evaluate F_5.

Assuming steady state, the effective outward CO_2 volumetric flux is given as,

$$J = F_{1,T} = 2F_{2,T} = 4F_{3,T} \quad (19)$$

where,

$$F_{3,T} = 4F_5 + 2F_5\frac{r_4L_4}{r_5L_5} + F_5\frac{r_3L_3}{r_5L_5} \quad (20)$$

References

1. Kestler P. Respiration and respiratory water loss. In: Hoffmann KH, editor. Environmental Physiology and Biochemistry of Insects (ed K. H. Hoffmann). Berlin: Springer; 1985. 137-183.
