# Supplementary material for: Tracheal branching in ants is area-decreasing, violating a central assumption of network transport models
Source: PLoS Comput Biol. 2020 Apr 30;16(4):e1007853. doi: 10.1371/journal.pcbi.1007853 (PMC7241831; doi:10.1371/journal.pcbi.1007853)
Supplement: S1 Table — (PDF) [file pcbi.1007853.s003.pdf]

Supporting Information S2

Tracheal branching in ants is area-decreasing, violating a central assumption of network transport models

Ian J. Aitkenhead[1], Grant A. Duffy[1], Citsabehsan Devendran[2], Michael R. Kearney[3], Adrian Neild[2] and Steven L. Chown[1,*]

1 School of Biological Sciences, Monash University, Victoria 3800, Australia, **2** Department of Mechanical and Aerospace Engineering, Monash University, Victoria 3800, Australia, **3** School of BioSciences, The University of Melbourne, Victoria 3010, Australia

* steven.chown@monash.edu

S2. Ant tracheal systems adhere to Nunome's pattern based on data from tracheal cross-sectional area measurements made in this study. Outcomes of the Ordinary Least Squares (OLS) regression forced through the intercept and Major Axis (Model II) regression for levels 1 to 2, and 2 to 3, in the ant abdominal tracheal system show that neither Da Vinci's rule, nor Murray's law apply to these systems. If they did, slopes of the relationships for each of the regressions within these categories should be approximately 1. They only assume this value for Nunome's pattern. In each case, the level mentioned first is considered X and the level mentioned second is considered Y in the regressions.

	**Slope Estimate ± s.e.**	**95% Confidence intervals**	**Significance**
**OLS**			
**Da Vinci's rule**			
Level 1 to 2	0.555 ± 0.016	0.523 – 0.587	$F_{(1, 164)} = 1179$, $p < 0.0001$, $R^2 = 0.88$
Level 2 to 3	0.526 ± 0.012	0.502 – 0.550	$F_{(1, 158)} = 1815$, $p < 0.0001$, $R^2 = 0.92$
Level 2 to 3 (b)	0.556 ± 0.021	0.514 – 0.598	$F_{(1, 152)} = 679$, $p < 0.0001$, $R^2 = 0.82$
**Murray's law**			
Level 1 to 2	0.277 ± 0.011	0.255 – 0.298	$F_{(1, 164)} = 643$, $p < 0.0001$, $R^2 = 0.80$
Level 2 to 3	0.306 ± 0.010	0.287 – 0.325	$F_{(1, 158)} = 1009$, $p < 0.0001$, $R^2 = 0.86$
Level 2 to 3 (b)	0.315 ± 0.022	0.270 – 0.357	$F_{(1, 152)} = 204$, $p < 0.0001$, $R^2 = 0.57$
**Nunome's pattern**			
Level 1 to 2	1.123 ± 0.021	1.081 – 1.165	$F_{(1, 164)} = 2791$, $p < 0.0001$, $R^2 = 0.94$
Level 2 to 3	0.984 ± 0.013	0.959 – 1.009	$F_{(1, 158)} = 5955$, $p < 0.0001$, $R^2 = 0.97$
Level 2 to 3 (b)	1.037 ± 0.016	1.006 – 1.068	$F_{(1, 152)} = 4455$, $p < 0.0001$, $R^2 = 0.97$

**Major Axis**	**Slope**	**95% C.I.**	**Significance (p)**
**Da Vinci's rule**			
Level 1 to 2	0.620	0.566 – 0.676	0.001
Level 2 to 3	0.558	0.523 – 0.593	0.001
Level 2 to 3 (b)	0.610	0.542 – 0.683	0.001
**Murray's law**			
Level 1 to 2	0.280	0.252 – 0.309	0.001
Level 2 to 3	0.319	0.296 – 0.342	0.001
Level 2 to 3 (b)	0.343	0.285 – 0.402	0.001
**Nunome's pattern**			
Level 1 to 2	1.527	1.400 – 1.672	0.001
Level 2 to 3	1.058	0.998 – 1.122	0.001
Level 2 to 3 (b)	1.085	1.003 – 1.175	0.001
