# Supplementary material for: Tracheal branching in ants is area-decreasing, violating a central assumption of network transport models
Source: PLoS Comput Biol. 2020 Apr 30;16(4):e1007853. doi: 10.1371/journal.pcbi.1007853 (PMC7241831; doi:10.1371/journal.pcbi.1007853)
Supplement: S2 Table — (PDF) [file pcbi.1007853.s004.pdf]

Supporting Information S3

Tracheal branching in ants is area-decreasing, violating a central assumption of network transport models

Ian J. Aitkenhead[1], Grant A. Duffy[1], Citsabehsan Devendran[2], Michael R. Kearney[3], Adrian Neild[2] and Steven L. Chown[1,*]

1 School of Biological Sciences, Monash University, Victoria 3800, Australia, **2** Department of Mechanical and Aerospace Engineering, Monash University, Victoria 3800, Australia, **3** School of BioSciences, The University of Melbourne, Victoria 3010, Australia

* steven.chown@monash.edu

S3. Outcomes of the linear mixed-effects model fit by restricted maximum likelihood with species as a random effect and the intercept forced through zero based on data collected for this study of ant tracheal cross-sectional area measurements. The outcomes show that even when including species identity, neither Da Vinci's rule, nor Murray's law apply to these systems. If they did, slopes of the relationships for each of the regressions within these categories should be approximately 1. They only assume this value, or a value very close to it, for Nunome's pattern. In each case, the level mentioned first is considered X and the level mentioned second is considered Y in the regressions.

	**Slope Estimate ± s.e.**	**95% Confidence intervals**	**Significance**
**Da Vinci's rule**			
Level 1 to 2	0.577 ± 0.023	0.531 - 0.623	$F_{(1, 145)} = 612.68$, $p < 0.0001$
Level 2 to 3	0.531 ± 0.015	0.500 - 0.561	$F_{(1, 139)} = 1179.58$, $p < 0.0001$
Level 2 to 3 (b)	0.566 ± 0.025	0.517 - 0.615	$F_{(1, 133)} = 523.24$, $p < 0.0001$
**Murray's law**			
Level 1 to 2	0.282 ± 0.014	0.254 - 0.310	$F_{(1, 145)} = 401.98$, $p < 0.0001$
Level 2 to 3	0.311 ± 0.011	0.289 - 0.333	$F_{(1, 139)} = 797.57$, $p < 0.0001$
Level 2 to 3 (b)	0.329 ± 0.025	0.279 – 0.378	$F_{(1, 133)} = 172.46$, $p < 0.0001$
**Nunome's pattern**			
Level 1 to 2	1.195 ± 0.040	1.116 - 1.274	$F_{(1, 145)} = 891.95$, $p < 0.0001$
Level 2 to 3	0.980 ± 0.019	0.941 - 1.018	$F_{(1, 139)} = 2544.51$, $p < 0.0001$
Level 2 to 3 (b)	1.036 ± 0.018	1.000 – 1.072	$F_{(1, 133)} = 3261.45$, $p < 0.0001$
