# Supplementary material for: Tracheal branching in ants is area-decreasing, violating a central assumption of network transport models
Source: PLoS Comput Biol. 2020 Apr 30;16(4):e1007853. doi: 10.1371/journal.pcbi.1007853 (PMC7241831; doi:10.1371/journal.pcbi.1007853)
Supplement: S3 Table — (PDF) [file pcbi.1007853.s005.pdf]

Supporting Information S6

Tracheal branching in ants is area-decreasing, violating a central assumption of network transport models

Ian J. Aitkenhead[1], Grant A. Duffy[1], Citsabehsan Devendran[2], Michael R. Kearney[3], Adrian Neild[2] and Steven L. Chown[1,*]

1 School of Biological Sciences, Monash University, Victoria 3800, Australia, **2** Department of Mechanical and Aerospace Engineering, Monash University, Victoria 3800, Australia, **3** School of BioSciences, The University of Melbourne, Victoria 3010, Australia

* steven.chown@monash.edu

S6. Previous measurements of the relationship between parent and child branches in insect tracheae. For some species multiple stages have been measured. In many cases no sample sizes or measures of variance are provided in the original investigations, suggesting that single individuals have been measured.

**Species**	**Order**	**Measures reported**	**Outcome**	**Relationship**	**Source**
**Immatures**					
Cossus larva	Lepidoptera	Actual parent and child diameters from a single larval preparation	Parent (D^2) 279, Child Σ d2 = 260	Area-preserving	Krogh 1920: 100
Bombyx mori larva	Lepidoptera	Actual tracheal diameters along full length	Cross-sectional area increasing then declining	Area-increasing then Area-reducing	Nunome 1951, as translated by Weis-Fogh 1964
Periplaneta sp. nymph	Blattodea	Ratio of sum of child cross-sectional area areas to parent area	0.99 ± 0.10 (SD), n = 28 forks measured	Area-preserving	Locke 1958: 375
Calliphora sp. larva	Diptera	Ratio of sum of child cross-sectional area areas to parent area	1.00 ± 0.13 (SD), n = 31 forks measured	Area-preserving	Locke 1958: 375
Rhodnius prolixus, 3rd instar nymph	Hemiptera	Ratio of sum of child cross-sectional area areas to parent area	1.00 ± 0.16 (SD), n = 25 forks measured	Area-preserving	Locke 1958: 375
Rhodnius prolixus, 4th instar nymph	Hemiptera	Ratio of sum of child cross-sectional area areas to parent area	1.00 ± 0.17 (SD), n = 25 forks measured	Area-preserving	Locke 1958: 375
Rhodnius prolixus, 5th instar nymph	Hemiptera	Ratio of sum of child cross-sectional area areas to parent area	0.97 ± 0.15 (SD), n = 24 forks measured	Area-preserving	Locke 1958: 375
Ephestia sp. larva	Lepidoptera	Ratio of sum of child cross-sectional area areas to parent area	1.02 ± 0.18 (SD), n = 31 forks measured	Area-preserving	Locke 1958: 375

**Adults**					
Aphelocheirus sp.	Hemiptera	Head musculature tracheae	Area increasing from 7.5 x 10^{-5} cm^2 to 9.2x 10^{-5} cm^2 and then declining to 7.3 x 10^{-5} cm^2	Area-increasing then Area-reducing	Thorpe & Crisp 1947: 285
Aphelocheirus sp.	Hemiptera	Tracheae from first thoracic spiracle	Area declining from 2.4 x 10^{-5} cm^2 to 1.7 x 10^{-5} cm^2 and then increasing to 2.8 x 10^{-5} cm^2	Area-reducing then Area-increasing	Thorpe & Crisp 1947: 285
Aphelocheirus sp.	Hemiptera	Tracheae from third thoracic spiracle	Area declining from 0.205 x 10^{-5} cm^2 to 0.136 x 10^{-5} cm^2	Area-reducing	Thorpe & Crisp 1947: 285
Aeshna sp.	Odonata	Lobe A of flight muscle	Declining diameter of secondary tube, but change to tertiary	Area-reducing	Weis-Fogh 1964: 237
Aeshna sp.	Odonata	Lobes B & C of flight muscle	Area reducing after first branching	Area-reducing	Weis-Fogh 1964: 238
Drosophila melanogaster	Diptera	Flight muscle	Diameter reducing	Area-reducing	Harrison et al. 2018: 195
Platynus decentis	Coleoptera	Tracheal system	Not Murray's law	~ Area-preserving	Socha et al. 2010; Kenny & Socha 2015

References

Harrison, J., Waters, J.S., Biddulph, T.A., Kovacevic, A., Klok, C.J. & Socha, J.J. (2018) Developmental plasticity and stability in the tracheal networks supplying *Drosophila* flight muscle in response to rearing oxygen level. *Journal of Insect Physiology* **106**, 189-198.

Kenny, M. & Socha, J.J. (2015) Does Murray's law apply to the tracheal system in insects? A 3D study of the beetle *Platynus decentis*. Society for Integrative and Comparative Biology Meeting Abstracts, p. 171. Available at: http://www.sicb.org/meetings/2015/SICB%20ABSTRACT%20BOOK.pdf (downloaded 13 March 2018)

Krogh, A. (1920) Studien über tracheenrespiration. II. Über gasdiffusion in den trachen. *Pflügers Archiv* **179,** 95-112.

Locke, M. (1958) The coordination of growth in the tracheal system of insects. *Quarterly Journal of the Microscopical Society* **99,** 373-391.

Nunome, J. (1951) Studies on the respiration of the silkworm. Part III. On the air current of respiration. *Journal of Sericological Science Japan* **20,** 111-127.

Socha, J.J., Forster, T.D. & Greenlee, K.J. (2010) Issues of convection in insect respiration: insights from synchrotron X-ray imaging and beyond. *Respiratory Physiology and Neurobiology* **173 Suppl,** S65-73.
Thorpe, W.H. & Crisp, D.J. (1947) Studies on plastron respiration II. The respiratory efficiency of the plastron in *Aphelocheirus*. *Journal of Experimental Biology* **24,** 270-303.
Weis-Fogh, T. (1964) Diffusion in insect wing muscle, the most active tissue known. *Journal of Experimental Biology* **41,** 229-256.
