# Supplementary material for: Tracheal branching in ants is area-decreasing, violating a central assumption of network transport models
Source: PLoS Comput Biol. 2020 Apr 30;16(4):e1007853. doi: 10.1371/journal.pcbi.1007853 (PMC7241831; doi:10.1371/journal.pcbi.1007853)
Supplement: S4 Table — (PDF) [file pcbi.1007853.s006.pdf]

Supporting Information S8

Tracheal branching in ants is area-decreasing, violating a central assumption of network transport models

Ian J. Aitkenhead[1], Grant A. Duffy[1], Citsabehsan Devendran[2], Michael R. Kearney[3], Adrian Neild[2] and Steven L. Chown[1,*]

1 School of Biological Sciences, Monash University, Victoria 3800, Australia, **2** Department of Mechanical and Aerospace Engineering, Monash University, Victoria 3800, Australia, **3** School of BioSciences, The University of Melbourne, Victoria 3010, Australia

* steven.chown@monash.edu

S8. Collection sites and number of specimens examined for each species in this study. Some species have both major and minor workers, and where this was the case the numbers of each collected are shown.

Species	Authority	Worker	Major worker	Minor worker	Location 1	Location 2	GPS locality 1	GPS locality 2	State	Reference
Camponotus aeneopilosus	Mayr, 1862	0	0	5	Kalimna Park, Castlemaine	na	-37.062024, 144.231451	na	Victoria	1
Camponotus aurocinctus	F. Smith, 1858	0	0	6	Jackass Flat Conservation Reserve	na	-36.726621, 144.285800	na	Victoria	1
Camponotus claripes	Mayr, 1876	0	8	10	Langeanook Track, Golden Gully, Bendigo	na	-36.793594, 144.272137	na	Victoria	1
Camponotus consobrinus	Erichson, 1846	0	0	5	Kalimna Park, Castlemaine	na	-37.062540, 144.231667	na	Victoria	1
Camponotus intrepidus	W. Kirby, 1819	0	2	7	Karawatha Forest	na	-27.64361111, 153.0733333	na	Queensland	1
Camponotus nigriceps	F. Smith, 1858	0	4	7	Kalimna Park, Castlemaine	na	-37.061371, 144.232982	na	Victoria	1
Camponotus oetkeri	Forel, 1910	0	8	4	Kings Billabong Park, Mildura	na	-34.251794, 142.231434	na	Victoria	1
Camponotus suffusus	F. Smith, 1858	0	6	7	Jackass Flat Conservation Reserve.	Kalimna Park, Castlemaine	-36.728897, 144.289000	-37.064012, 144.230092	Victoria	1

Irydomermex purpureus	F. Smith, 1858	13	0	0	Jackass Flat Conservation Reserve	Kings Billabong Park, Mildura	-36.728804, 144.282794	-34.28395, 142.241534	Victoria	2
Myrmecia brevinoda	Forel, 1910	13	0	0	Clayton, Melbourne	na	-37.908601, 145.138882	na	Victoria	3
Myrmecia forficata	Fabricuius, 1787	3	0	0	Kalimna Park, Castlemaine	na	-37.062253, 144.230163	na	Victoria	3
Myrmecia fulvipes	Roger, 1861	7	0	0	Kalimna Park, Castlemaine	na	-37.052305, 144.236289	na	Victoria	3
Myrmecia pyriformis	F. Smith, 1858	7	0	0	Langeanook Track, Golden Gully, Bendigo	na	-36.802011, 144.269540	na	Victoria	3
Polyrhachis ammon	Fabricuius, 1775	7	0	0	Kangaroo Point, Brisbane	na	-27.474, 153.0354	na	Queensland	4
Rhytidoponera chalybaea	Emery, 1901	7	0	0	Roma Parklands	na	-27.6425,153.0713889	na	Queensland	5
Rhytidoponera mayri	Emery, 1883	7	0	0	Kalimna Park, Castlemaine	na	-37.063010, 144.231997	na	Victoria	6
Rhytidoponera metallica	F. Smith, 1858	7	0	0	Kings Billabong Park, Mildura	na	-34.28395, 142.231534	na	Victoria	7
Rhytidoponera nodifera	Emery, 1895	6	0	0	Toohey Forest	na	-27.54, 153.025	na	Queensland	6

Rhytidoponera punctiventris	Forel, 1900	7	0	0	Kalimna Park, Castlemaine	na	-37.055447, 144.234340	na	Victoria	6
Rhytidoponera victoriae	Andre, 1896	5	0	0	Toohey Forest	na	-27.54, 153.025	na	Queensland	8

References

1. McArthur, A. J. 2007. A key to *Camponotus* Mayr of Australia. Pages 290-351 in Snelling, R. R., B. L. Fisher and P. S. Ward. Advances in ant systematics (Hymenoptera: Formicidae): Homage to E.O. Wilson - 50 years of contributions. Memoirs of the American Entomological Institute, vol. 80.

2. Heterick, B. E. & Shattuck, S. (2011) Revision of the ant genus *Iridomyrmex* (Hymenoptera: Formicidae). Zootaxa 2845: 1-174.

3. Ogata, K., Taylor, R.W. (1991) Ants of the genus *Myrmecia* Fabricius: a preliminary review and key to the named species (Hymenoptera: Formicidae: Myrmeciinae). Journal of Natural History, 25, 1623–1673.

4. Kohout, R.J. 2013. Revision of *Polyrhachis* (*Hagiomyrma*) Wheeler, 1911 (Insecta: Hymenoptera: Formicidae: Formicinae). Memoirs of the Queensland Museum, Nature 56, 487-577.

5. Ward, P. S. 1980a. A systematic revision of the *Rhytidoponera impressa* group (Hymenoptera: Formicidae) in Australia and New Guinea. Australian Journal of Zoology 28: 475-498

6. Clark, J. 1936. A revision of Australian Species of *Rhytidoponera mayr* (Formicidae). Memoirs of the National Museum of Victoria 9. 14-89.

7. Crawley, W.C. 1925b. New Ants from Australia. - II. Annals and magazine of Natural History. 9(16): 577-598.

8. Crawley, W. C. 1922d. Notes on some Australian ants. Biological notes by E. B. Poulton, D.Sc., M.A., F.R.S., and notes and descriptions of new forms by W. C. Crawley, B.A., F.E.S., F.R.M.S. [concl.]. Entomologists Monthly Magazine 58: 121-126
