# Supplementary material for: Tracheal branching in ants is area-decreasing, violating a central assumption of network transport models
Source: PLoS Comput Biol. 2020 Apr 30;16(4):e1007853. doi: 10.1371/journal.pcbi.1007853 (PMC7241831; doi:10.1371/journal.pcbi.1007853)
Supplement: S5 Table — (PDF) [file pcbi.1007853.s007.pdf]

Supporting Information S9

Tracheal branching in ants is area-decreasing, violating a central assumption of network transport models

Ian J. Aitkenhead[1], Grant A. Duffy[1], Citsabehsan Devendran[2], Michael R. Kearney[3], Adrian Neild[2] and Steven L. Chown[1,*]

1 School of Biological Sciences, Monash University, Victoria 3800, Australia, **2** Department of Mechanical and Aerospace Engineering, Monash University, Victoria 3800, Australia, **3** School of BioSciences, The University of Melbourne, Victoria 3010, Australia

* steven.chown@monash.edu

S9. Published empirical data on ant metabolic rates used to estimate CO_2 flux for the whole animal. See methods on details of the approach used to extract the data and make conversions as required.

**Species**	**Metabolic rate (ml/s)**	**State**	**Temperature (deg C)**	**Mass (mg)**	**MR (ul/h)**	**Source**
Paraponera clavata	1.64E-04	running 0.07 m.s-1	28	200	591.30	Fewell et al. 1996
Solenopsis invicta female	1.43E-04	flight	33.6	15.2	513.76	Vogt et al. 2000
Solenopsis invicta male	9.65E-05	flight	32.6	7.3	347.48	Vogt et al. 2000
Paraponera clavata	5.33E-05	active exploring	28	200	192.00	Fewell et al. 1996
Paraponera clavata	5.08E-05	active exploring	28	200	182.96	Fewell et al. 1996
Camponotus detritus	1.59E-05	rest	30	69.2	57.37	Lighton 1990
Pogonomyrmex rugosus	1.20E-05	running 0.07 m.s-1	43	16.5	43.04	Lighton & Feener 1989
Atta columbica	1.17E-05	running unladen	28	15.6	42.12	Lighton et al. 1987
Camponotus fulvopilosus	9.56E-06	rest	40	43	34.40	Lighton 1989
Eciton hamatum	8.39E-06	running	28	10	30.20	Bartholomew et al. 1988
Camponotus sp.	7.55E-06	walking	27	11.9	27.16	Lipp et al. 2005
Formica rufa	6.71E-06	rest	23	11.1	24.15	Perl & Niven 2018
Myrmecocystus mendax	5.27E-06	laden running	40	6.19	18.98	Duncan & Lighton 1994
Formica rufa	5.03E-06	rest	23	11.1	18.12	Perl & Niven 2018
Camponotus detritus	4.97E-06	rest	30	44.4	17.87	Lighton 1990
Myrmecocystus mendax	4.00E-06	unladen running	40	6.04	14.41	Duncan & Lighton 1994
Pogonomyrmex occidentalis	3.09E-06	walking	36	6.02	11.11	Fewell 1988
Camponotus vicinus	2.79E-06	rest	25	35.15	10.06	Lighton 1992
Eciton hamatum	1.91E-06	rest	28	10	6.89	Bartholomew et al. 1988
Pogonomyrmex rugosus worker	1.91E-06	rest	40	15	6.88	Lighton & Bartholomew 1988

Messor pergandei female alate	1.79E-06	rest	24	39.4	6.46	Lighton & Berrigan 1995
Camponotus maculatus	1.51E-06	rest	20	41.376	5.43	Chown et al. 2007
Pogonomyrmex rugosus female alate	1.42E-06	rest	25	32.2	5.12	Lighton et al. 1993
Messor pergandei male alate	1.38E-06	rest	24	16.47	4.98	Lighton & Berrigan 1995
Camponotus sp.	1.28E-06	rest	27	11.9	4.60	Lipp et al. 2005
Cataglyphis bicolor	1.19E-06	rest	25	24.5	4.29	Lighton 1992
Myrmecocystus mendax	1.13E-06	rest	40	6.04	4.08	Duncan & Lighton 1994
Messor capensis	1.12E-06	rest	20	13.75	4.03	Chown et al. 2007
Messor julianus female alate	9.72E-07	rest	24	21.11	3.50	Lighton & Berrigan 1995
Messor pergandei worker	5.42E-07	rest	24	7.19	1.95	Lighton & Berrigan 1995
Anoplolepis steinergroevei	3.56E-07	rest	20	5.536	1.28	Chown et al. 2007
Messor julianus worker	2.75E-07	rest	24	5.09	0.99	Lighton & Berrigan 1995
Camponotus capito	2.26E-07	rest	20		0.82	Chown et al. 2007
Cataglyphis bicolor	2.01E-07	rest	40	34	0.73	Lighton & Wehner 1993
Camponotus suffusus	1.94E-07	rest	20		0.70	Chown et al. 2007
Camponotus consobrinus	1.75E-07	rest	20		0.63	Chown et al. 2007
Atta columbica	1.11E-08	rest	28	10	0.04	Lighton et al. 1987

References

Bartholomew, G.A., Lighton, J.R.B. & Feener, D.H. (1988) Energetics of trail running, load carriage, and emigration in the column-raiding army ant *Eciton hamatum*. *Physiological Zoology* **61**, 57-68.

Chown, S.L. et al. (2007) Scaling of insect metabolic rate is inconsistent with the nutrient supply network model. *Functional Ecology* **21**, 282-290.

Duncan, F.D. & Lighton, J.R.B. (1994) The burden within: the energy cost of load carriage in the honeypot ant, *Myrmecocystus. Physiological Zoology* **67**, 190-203.

Fewell, J.H. (1988) Energetic and time costs of foraging in harvester ants, *Pogonomyrmex occidentalis*. *Behavioural Ecology and Sociobiology* **22**, 401-408 (1988).

Fewell, J.H., Harrison, J.F., Lighton, J.R.B. & Breed, M.D. (1996) Foraging energetics of the ant, *Paraponera clavata*. *Oecologia* **105**, 419-427.

Lighton, J.R.B. (1989) Individual and whole-colony respiration in an African formicine ant. *Functional Ecology* **3**, 523-530.

Lighton, J.R.B. (1990) Slow discontinuous ventilation in the Namib dune-sea ant *Camponotus detritus* (Hymenoptera, Formicidae). *Journal of Experimental Biology* **151**, 71-82.

Lighton, J.R.B. (1992) Direct measurement of mass loss during discontinuous ventilation in two species of ants. *Journal of Experimental Biology* **173**, 289-293.

Lighton, J.R.B. & Bartholomew, G.A. (1988) Standard energy metabolism of a desert harvester ant, *Pogonomyrmex rugosus*: effects of temperature, body mass, group size, and humidity. *Proceedings of the National Academy of Sciences of the USA* **85**, 4765-4769.

Lighton, J.R.B., Bartholomew, G.A. & Feener, D.H. (1987) Energetics of locomotion and load carriage and a model of the energy cost of foraging in the leaf-cutting ant *Atta colombica* Guer. *Physiolological Zoology* **60**, 524-537.

Lighton, J.R.B. & Berrigan, D. (1995) Questioning paradigms: caste-specific ventilation in harvester ants, *Messor pergandei* and *M. julianus* (Hymenoptera: Formicidae). *Journal of Experimental Biology* **198**, 521-530.

Lighton, J.R.B. & Feener, D.H. (1989) A comparison of energetics and ventilation of desert ants during voluntary and forced locomotion. *Nature* **342**, 174-175.

Lighton, J.R.B. & Wehner, R. (1993) Ventilation and respiratory metabolism in the thermophilic desert ant, *Cataglyphis bicolor* (Hymenoptera, Formicidae). *Journal of Comparative Physiology B* **163**, 11-17.

Lighton, J.R.B., Weier, J.A. & Feener, D.H. (1993) The energetics of locomotion and load carriage in the desert harvester ant *Pogonomyrmex rugosus*. *Journal of Experimental Biology* **181**, 49-61.

Lipp, A., Wolf, H. & Lehmann, F.O. (2005) Walking on inclines: energetics of locomotion in the ant *Camponotus*. *Journal of Experimental Biology* **208**, 707-719.

Perl, C.D. & Niven, J.E. (2018) Metabolic rate scaling, ventilation patterns and respiratory water loss in red wood ants: activity drives ventilation changes, metabolic rate drives water loss. *Journal of Experimental Biology* **221**, jeb182501.

Vogt, J.T., Appel, A.G. & S. West, M. (2000) Flight energetics and dispersal capability of the fire ant, *Solenopsis invicta* Buren. *Journal of Insect Physiology* **46**, 697-707.
