# Supplementary material for: Tracheal branching in ants is area-decreasing, violating a central assumption of network transport models
Source: PLoS Comput Biol. 2020 Apr 30;16(4):e1007853. doi: 10.1371/journal.pcbi.1007853 (PMC7241831; doi:10.1371/journal.pcbi.1007853)
Supplement: S1 Fig — (PDF) [file pcbi.1007853.s008.pdf]

Supporting Information S5

Tracheal branching in ants is area-decreasing, violating a central assumption of network transport models

Ian J. Aitkenhead[1], Grant A. Duffy[1], Citsabehsan Devendran[2], Michael R. Kearney[3], Adrian Neild[2] and Steven L. Chown[1,*]

1 School of Biological Sciences, Monash University, Victoria 3800, Australia, **2** Department of Mechanical and Aerospace Engineering, Monash University, Victoria 3800, Australia, **3** School of BioSciences, The University of Melbourne, Victoria 3010, Australia

* steven.chown@monash.edu

S5. Effect of altered *pp*CO_2 gradient on modelled whole-organism CO_2 flux. Results for area-conserving (yellow line), area-increasing (orange line), or area-reducing (black lines) transport networks shown. Each of the three black lines represents a model system with either no air sac (continuous solid line) or an air sac at either the 3rd or 4th level (indicated by the dashed line-segment). Tracheal branch lengths of the modelled system ranged from 211 µm at the deepest level (level 5) to 1569 µm at level 1 (levels 4, 3, 2 had branch lengths of 352, 586, and 976 µm, respectively). The tracheal radius of level 5 was set at 3 µm. Whole organism flux was modelled with a partial pressure difference of 4, 6, or 10 kPa and assuming 6 pairs of abdominal spiracles. Horizontal grey lines indicate the flux needed to meet the metabolic requirements of an average ant at rest or walking (Supporting Information S9).

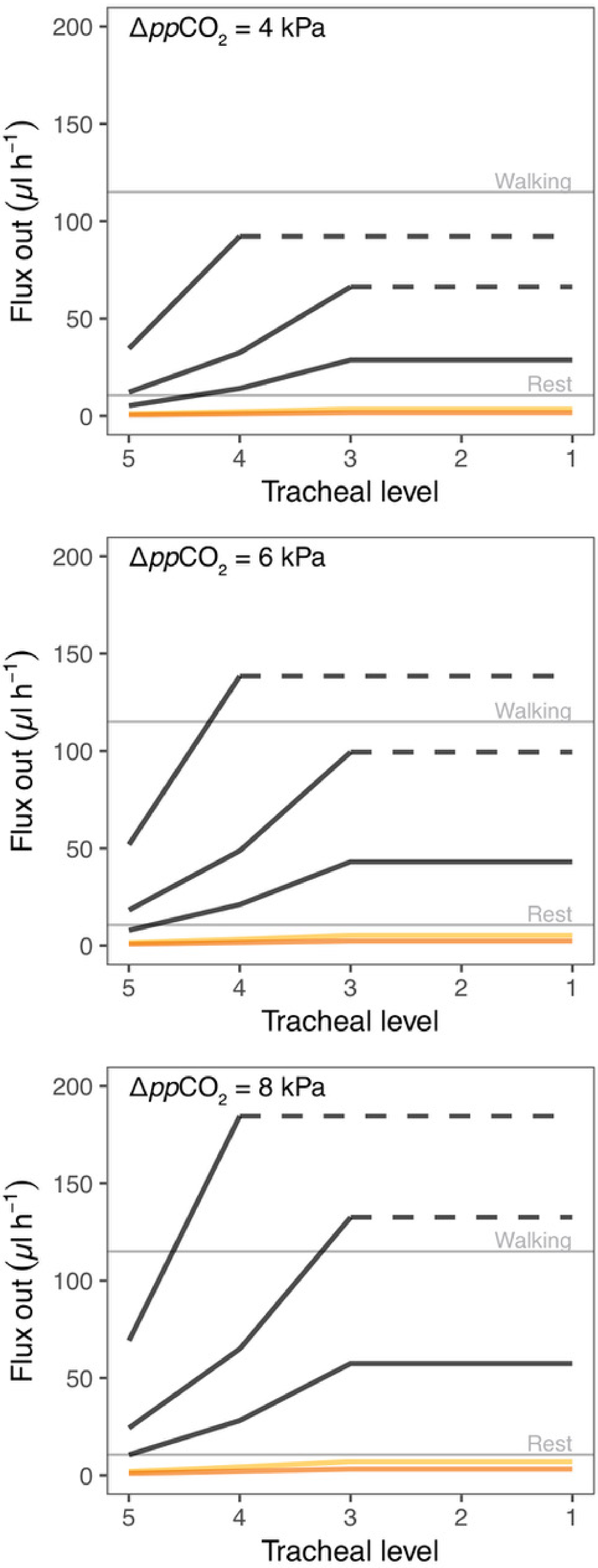

Δ*pp*CO_2 = 4 kPa
Δ*pp*CO_2 = 6 kPa
Δ*pp*CO_2 = 8 kPa
Walking
Rest
Flux out (μl h^{-1})
0
50
100
150
200
5
4
3
2
1
Tracheal level
