# Supplementary material for: Tracheal branching in ants is area-decreasing, violating a central assumption of network transport models
Source: PLoS Comput Biol. 2020 Apr 30;16(4):e1007853. doi: 10.1371/journal.pcbi.1007853 (PMC7241831; doi:10.1371/journal.pcbi.1007853)
Supplement: S2 Fig — (PDF) [file pcbi.1007853.s009.pdf]

Supporting Information S7

Tracheal branching in ants is area-decreasing, violating a central assumption of network transport models

Ian J. Aitkenhead[1], Grant A. Duffy[1], Citsabehsan Devendran[2], Michael R. Kearney[3], Adrian Neild[2] and Steven L. Chown[1,*]

1 School of Biological Sciences, Monash University, Victoria 3800, Australia, **2** Department of Mechanical and Aerospace Engineering, Monash University, Victoria 3800, Australia, **3** School of BioSciences, The University of Melbourne, Victoria 3010, Australia

* steven.chown@monash.edu

S7. Time-calibrated phylogenetic tree for all ant species examined. Derived from Economo et al.'s (2018, *Nature Communications* **9**, 1778) median tree with absent species placed by association with a randomly selected congeneric.

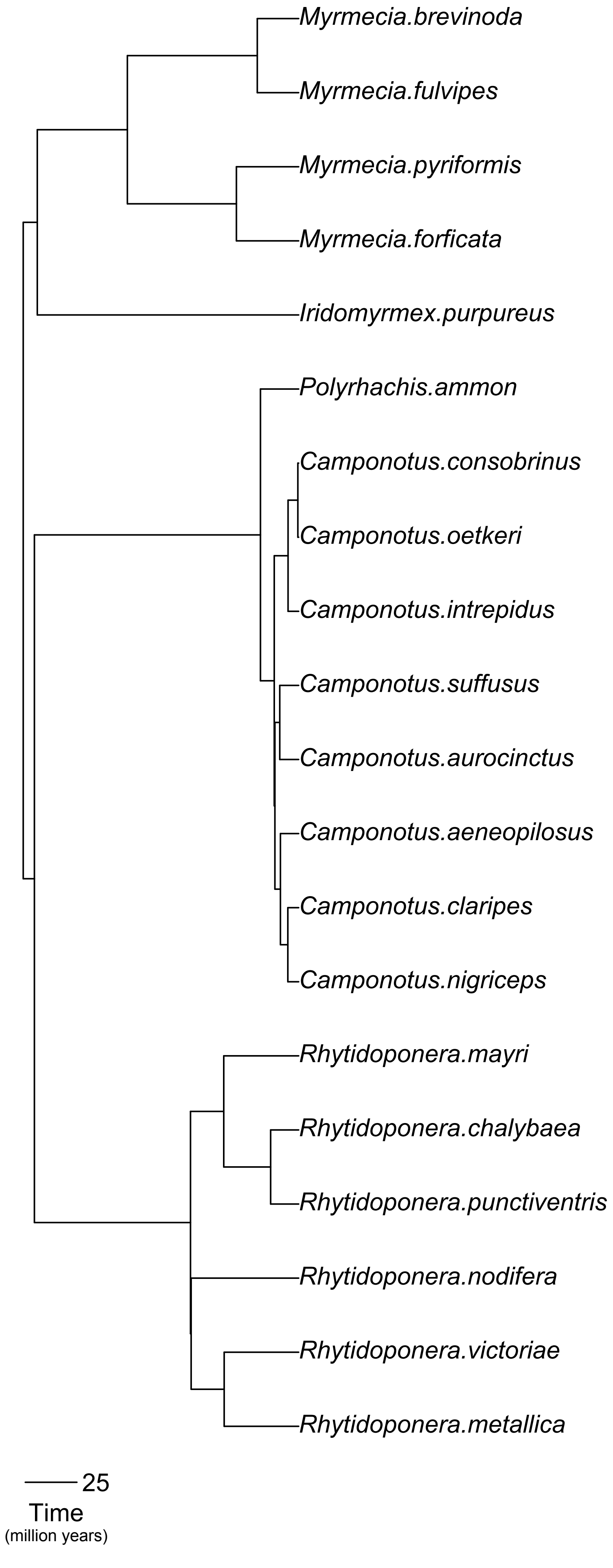

Myrmecia.brevinoda
Myrmecia.fulvipes
Myrmecia.pyriformis
Myrmecia.forficata
Iridomyrmex.purpureus
Polyrhachis.ammon
Camponotus.consobrinus
Camponotus.oetkeri
Camponotus.intrepidus
Camponotus.suffusus
Camponotus.aurocinctus
Camponotus.aeneopilosus
Camponotus.claripes
Camponotus.nigriceps
Rhytidoponera.mayri
Rhytidoponera.chalybaea
Rhytidoponera.punctiventris
Rhytidoponera.nodifera
Rhytidoponera.victoriae
Rhytidoponera.metallica
25
Time
(million years)
