# Supplementary material for: Tracheal branching in ants is area-decreasing, violating a central assumption of network transport models
Source: PLoS Comput Biol. 2020 Apr 30;16(4):e1007853. doi: 10.1371/journal.pcbi.1007853 (PMC7241831; doi:10.1371/journal.pcbi.1007853)

Supporting Information S10

Tracheal branching in ants is area-decreasing, violating a central assumption of network transport models

Ian J. Aitkenhead[1], Grant A. Duffy[1], Citsabehsan Devendran[2], Michael R. Kearney[3], Adrian Neild[2] and Steven L. Chown[1],*

1 School of Biological Sciences, Monash University, Victoria 3800, Australia, 2 Department of Mechanical and Aerospace Engineering, Monash University, Victoria 3800, Australia, 3 School of BioSciences, University of Melbourne, Victoria 3010, Australia

Raw, annotated synchrotron x-ray tomography slices from an individual of each genus investigated

A series of enlarged raw slices through the abdomen of individuals, all images are converted to 8-Bit and have had their contrast enhanced and have been annotated. These are the only manipulations.

Image **S10A.** is a *Camponotus consmobrinus* major caste. **S10B.** is a *Camponotus consmobrinus* minor caste. **S10C.** is an *Irydomermex purpureus* **S10D.** is a *Myrmercia fulvipes* **S10E.** is a *Rhytidoponera metallica* and **S10F.** is a *Polyrhachis amnon*.

Annotations are the same throughout; S is the mounting syringe. AS is an air sac. G is the gut of the animal, CG is the chemical gland. Trachea are denoted by a single white vertical line above the structure. Trachea are generally round in appearance in these slices, there are occasions where they appear as oval or line type structures, this is a visual artefact of caused by the tube intersecting the 2D plain at different angles. The scale-bar is consistent throughout at 500µm.

S10A. Camponotus consmobrinus (Major Caste).

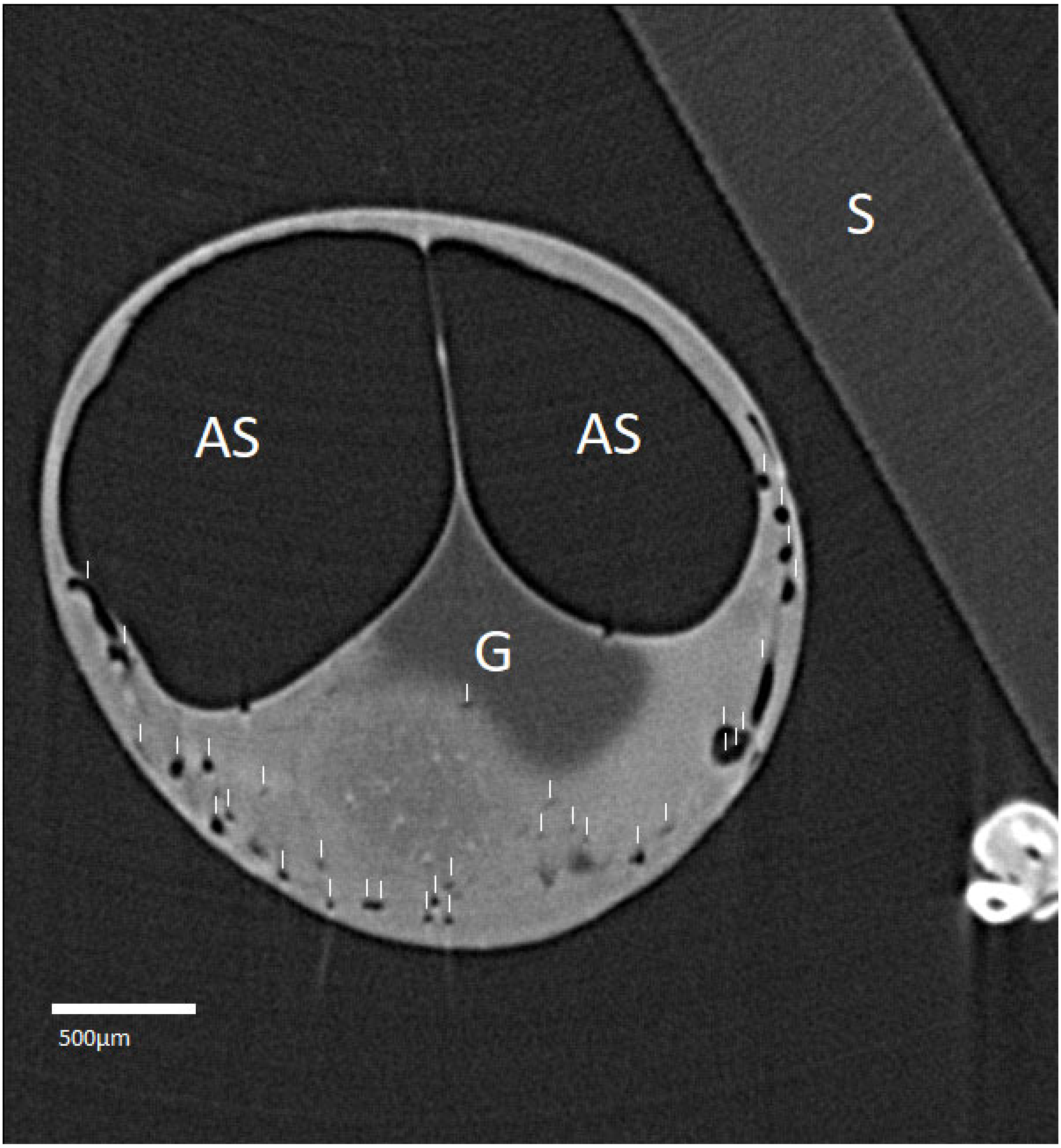

S10B. *Camponotus consmobrinus* (minor caste). Note in this image the orientation of the individual is such that the 2D planar is cutting lengthways through the abdomen rather than across. Because of this only a single air sac is present.

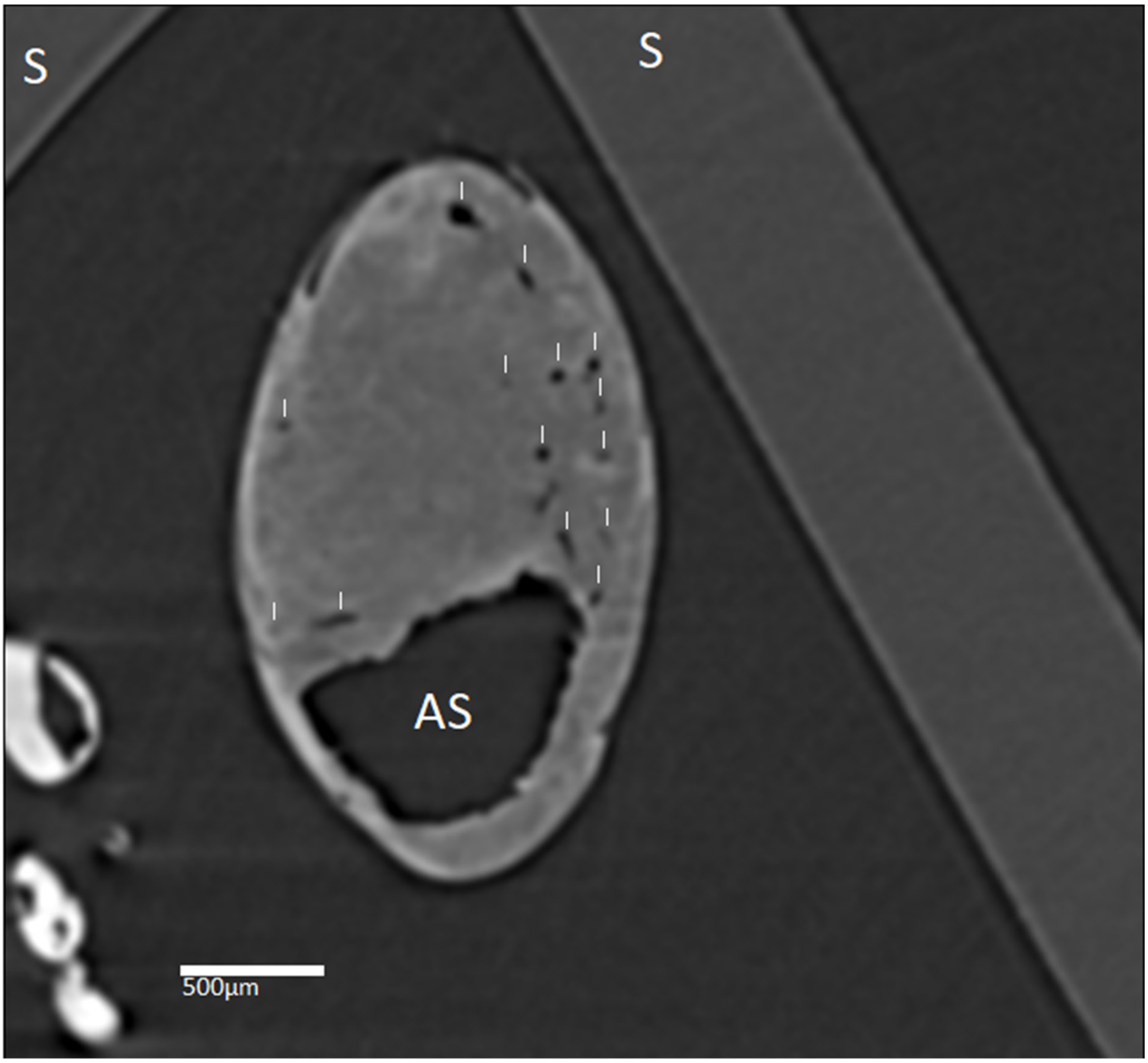

S10C. *Irydomermex purpureus*. Note the lack of air sacs in this image is due to the slice coming from a region below where the air sacs are present in the abdomen.

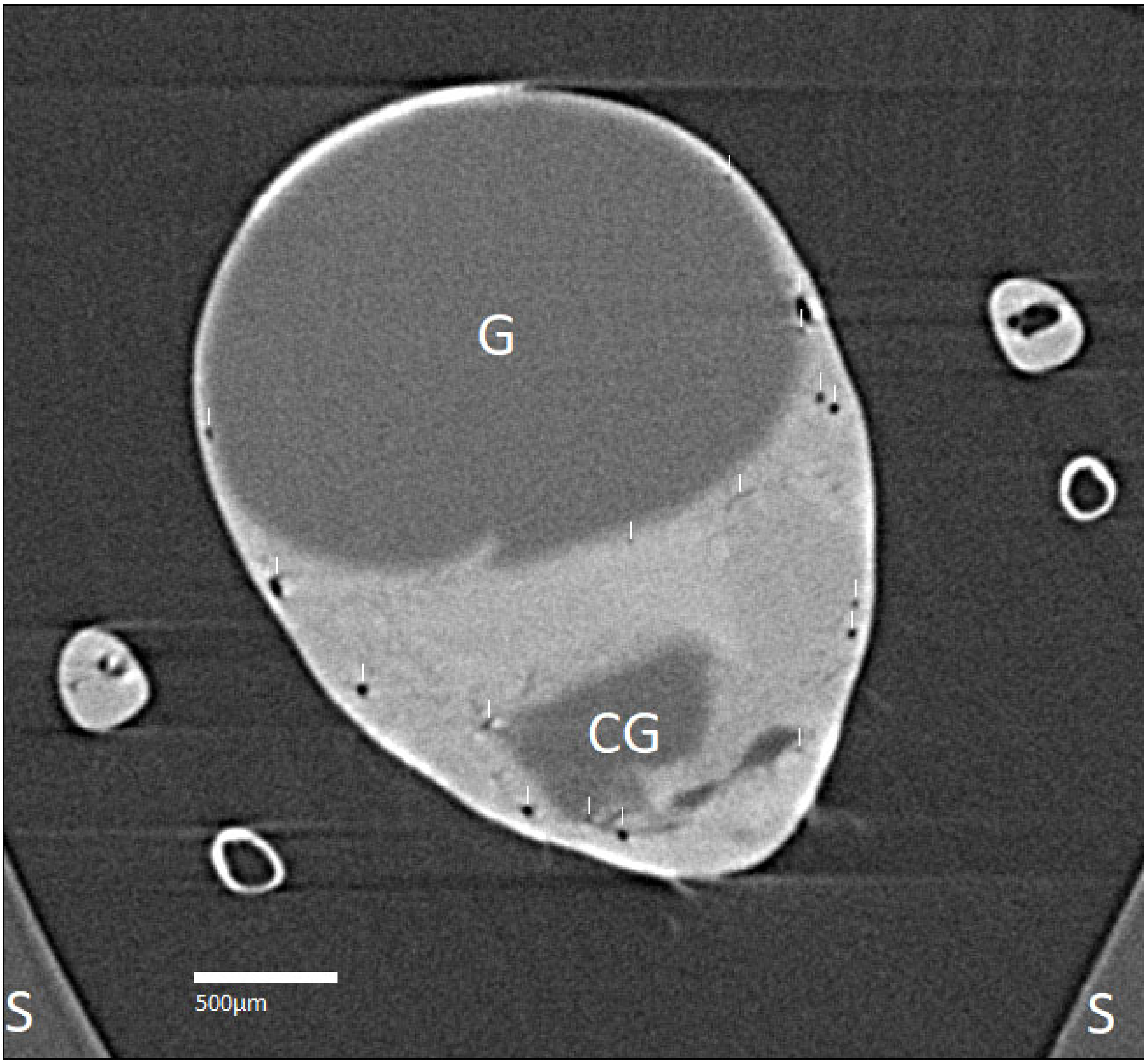

S10D. *Myrmecia fulvipres*. Note the appearance of multiple air sacs, qualitatively this was something associated with species that had a stinging structure present in the abdomen. The empty large unlabelled airspace at the bottom of the image is found in all individuals of the genus *Myrmecia* and forms around the stinger, this is an isolated airspace, separate to the tracheal system.

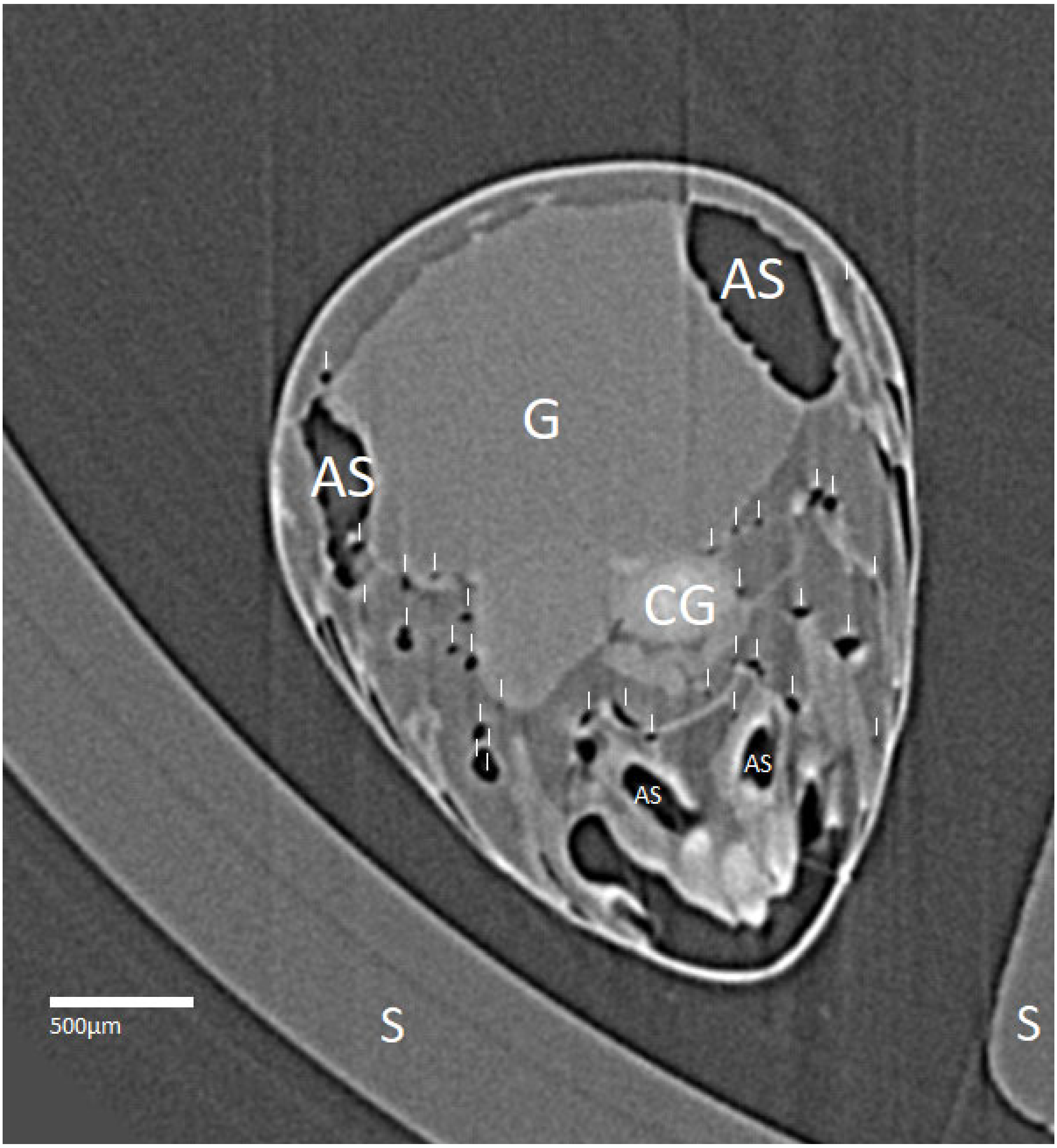

S10E. *Rhytidoponera metallica.* Note the appearance of multiple air sacs, qualitatively this was something associated with species that had a stinging structure present in the abdomen.

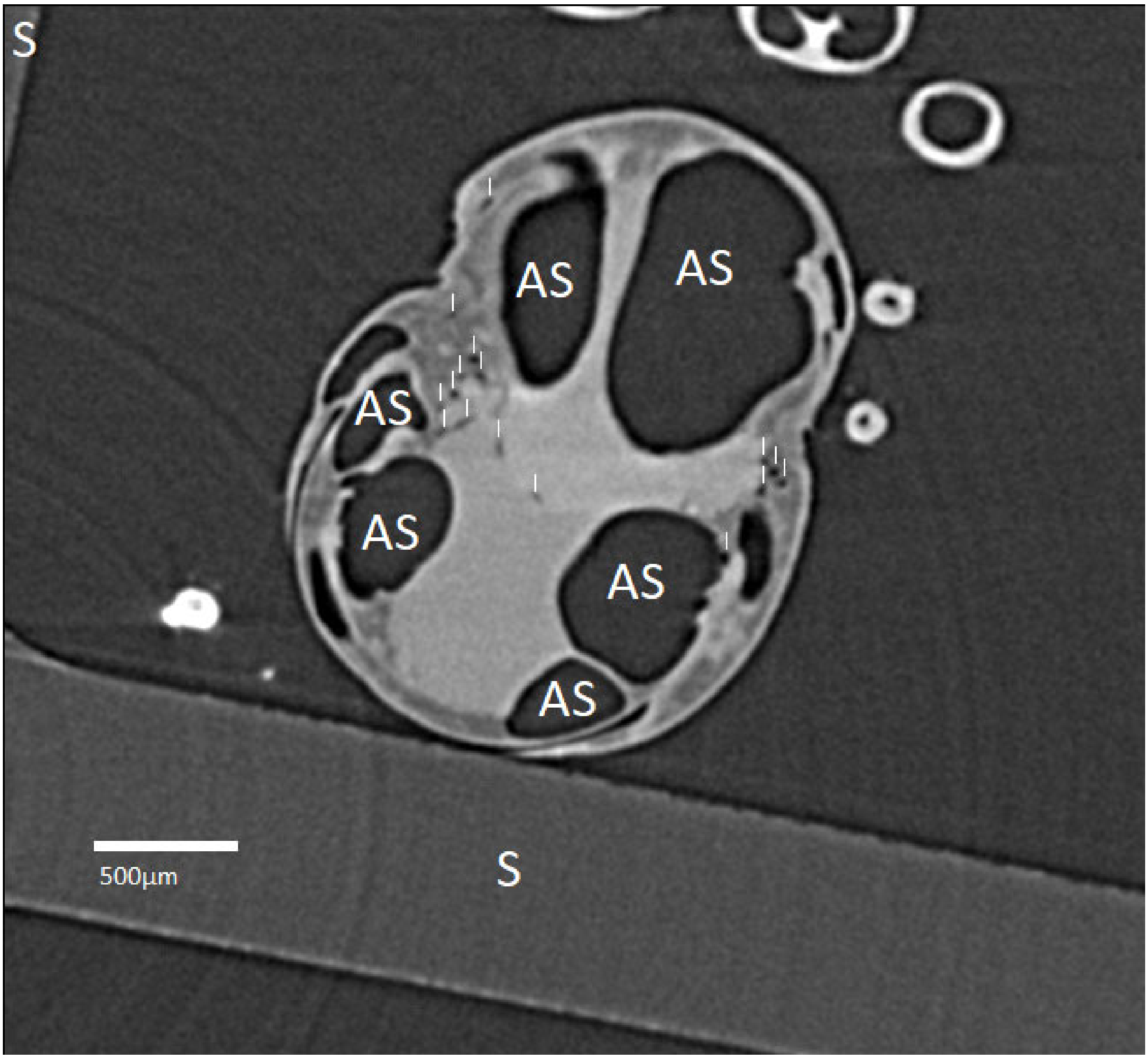

S10F. *Polyrhachis ammon*. Note here the appearance of a single air sac is due to the abdomen of the animal intersecting the planar on an angle. The head and legs of the individual are present but unlabelled through the middle and bottom of the image also.

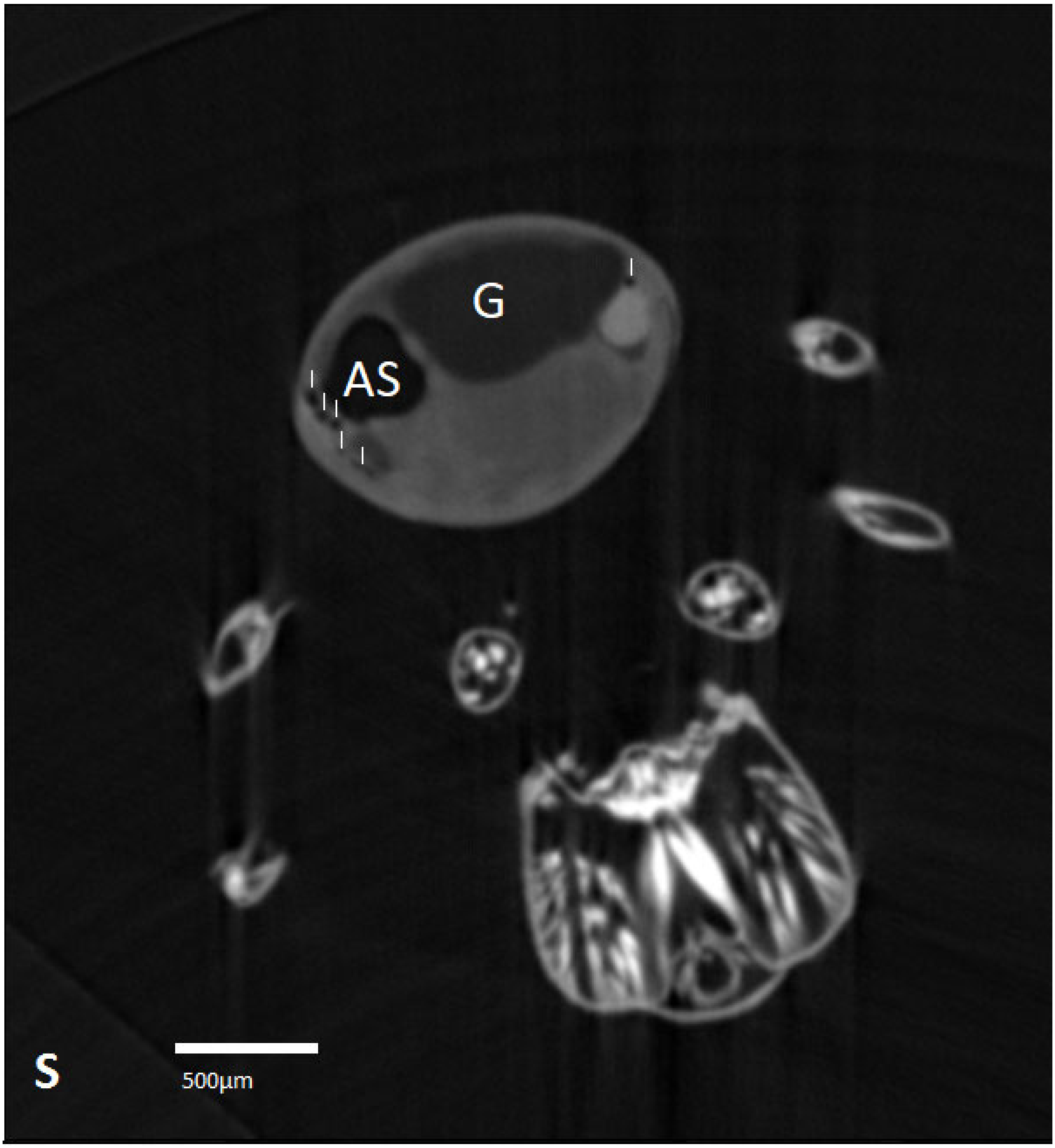

Supplement: S3 Fig — (PDF) [file pcbi.1007853.s010.pdf]
