# Supplementary material for: Tracheal branching in ants is area-decreasing, violating a central assumption of network transport models
Source: PLoS Comput Biol. 2020 Apr 30;16(4):e1007853. doi: 10.1371/journal.pcbi.1007853 (PMC7241831; doi:10.1371/journal.pcbi.1007853)
Supplement: Attachment — Submitted filename: my review.pdf [file pcbi.1007853.s011.pdf]

This manuscript is a truly exciting piece of work. Ever since the first high-profile papers of the (poorly named) metabolic theory of ecology came out, I have scratched my head over the claims that insect tracheal systems conform to their hypothesis. There were so little data on tracheal branching patterns in insects (the compilation in S6 documents this well; it’s great to see), and so many species, and so much variation, that really insects should have been considered as an unknown. The current work examines branching patterns in 20 species of insects and finds a surprising result: that cross-sectional area decreases down the network. Additional modeling adds another surprise: that CO_2 is really the culprit, not O_2. Just fascinating. And I would note that while collecting synchrotron tomography data is relatively fast (compared to classical techniques), the data analysis is not fast at all. The data and analysis presented must have taken a very large effort, and I hope that that is not lost on readers. Overall, I think that this is a very important manuscript, and it should have a large impact in the literature.

Despite my great enthusiasm, I have many specific comments for improvement of the manuscript, documented below. And I have two general comments:

1) The language about what the exponents ‘should’ be is not quite right. There is not just one set of values that provide area constant or area increasing characteristics; other values are possible. (In fact we find intermediate values in the beetle we studied, in a manuscript that is soon to be submitted.)

2) The supplements didn’t seem to be drafted with the same level of care as the rest of the manuscript, and need improvement. (Details below.)

-Jake Socha, Virginia Tech

Page 2:

>“The assumption of such a pattern underpins one of biology’s most influential ideas – the metabolic theory of ecology.”

Add a citation for the latter part of the sentence.

Page 3:

“with a transition to an increase in total cross-sectional area of smaller vessels (also known as Murray’s law) [1,8]”

>“Thus, the summed cross-sectional area of the child branches is either the same as or larger than the cross-sectional area of the parent branch (Fig 1).”

Given that the previous sentence discussed several options, using “thus” does not make sense here.

Page 4:

>“What the situation is for insects remains poorly known, resting, in discussions of metabolic scaling, on early empirical work [12] suggesting that branching is area-preserving (based on a single individual insect; Supporting Information S1), and on an assumption that diffusion dominates gas exchange through the tracheal system [1,13], which is the main transport route for O_2 to insect tissues and for CO_2 efflux from the body [14].”

This long sentence is a bit disappointing given the clear, elegant argument that was made in the cover letter. I would suggest expanding this a bit and borrowing simpler language from the letter, which is far more compelling.

>“Specifically, we tested the prediction that, in the equation relating parent to child branches:
$r\ r\ r$ (1)
α should be 2 or 3, as is the case for area-preserving and area-increasing branching patterns,”

There’s something off about the language here, but I can’t quite articulate what it is. There is no reason why alpha should be 2 or 3 in insect tracheal systems, unless there is a specific hypothesis of function. The condition where cost of flow and investment in wall material are both minimized, alpha is 3, but that does not mean that alpha should be 3; if it were found, it would suggest that evolution drove branching patterns in that direction.

Page 6

>“Using tracheal network data from our investigations, along with partial pressure variation data from the literature [20,22], the modelling indicates that tracheal architecture with an inwards decline in cross-sectional area with branching (which means area-increasing branching outwards), delivers the highest potential CO2 flux, in keeping with empirical data on ant metabolic rates (Fig 3c; Supporting Information S5).”

This sentence could be split up to help improve clarity.

>“Thus, for the 30 species of insects that have been examined for cross-sectional area variation among…”

This ‘thus’ doesn’t fit here, given the previous sentence.

Page 7

>“Hereafter we term a pattern of area reduction with branching ‘Nunome’s pattern’, acknowledging his early recognition thereof [26].”

I was thrown off here, because I felt like I missed something earlier about Nunome. This is the first mention of his name, so perhaps the explanation should come before the naming of the pattern.

>“In consequence, such a transport network should provide a biophysical advantage proximally, and ultimately a fitness benefit.”

I think that ‘physiological’ is more appropriate than ‘biophysical’; the former encompasses the latter.

>“In consequence tracheal systems are likely to be optimised for CO2 transport outwards.”

In the context of this paragraph, ‘specialized’ might be more appropriate than ‘optimised’, which is difficult to prove in an evolutionary sense. Also, add a comma after ‘consequence’.

>“accounting for the empirical data demonstrating that Murray’s law is rare in insects (Supporting Information S6).”

S6 seems to indicate that Murray’s law has not been found in insects. Our study (cited as an abstract) is about to be submitted for review and is might be better cited here as in review, if allowed. If not, Kenny’s 2019 PhD thesis (“Structural and physical characterization of insect flow systems”) is a better reference.

Page 8

>“Our data for the more proximal tracheal branches also show that such branching patterns are not simply restricted to more distal elements of the system as suggested recently [27].”

I don’t seem to remember a statement to that effect in that paper, but I could be wrong.

>Regarding the capitalization of ‘x-ray’: Although there is some debate about style, “x-ray” is probably better depicted without the capital letter. This is a minor matter, but see here for debate: https://english.stackexchange.com/questions/9891/should-the-x-in-x-ray-be-capitalized/9899#9899

>“Irrespective, two major empirical assumptions of transport network models, that branching is either area-preserving…”

There are loads of network transport models in the biological and engineering literature. Be specific here.

>“They were then exposed to iodine vapour for 7 days to improve contrast for imaging [37], which resulted in death.”

I’m very interested in this technique and will try it myself, but I wonder why it was needed in the first place. Generally with x-ray tomography (both synchrotron and tabletop), the contrast between air and tissue is quite high, and there’s no real need to do anything to the tissue. If you look at the first synchrotron tomography images of insect tracheal systems made in the mid 2000’s, the contrast was quite high. Overall, I’m somewhat surprised by the slice images shown in figure 2, but they are good enough for the analysis.

For reference, the referred images are in the following paper (which is obscure but can be downloaded from my lab’s website): Socha, J. J. and F. De Carlo (2008). Use of synchrotron tomography to image naturalistic anatomy in insects. Developments in X-Ray Tomography VI: 2008, San Diego, CA, SPIE, 70780A-70787.

>“in preparation for Synchrotron X-Ray tomography”

No capitals needed.

Page 10

>More details needed. What was the exposure time per image, and how long did a scan take to collect? This duration has an impact on ability to control motion in the tracheal system (induced by heating or other things). How many scans per insect? What was the field of view for the raw projections images? Was vertical stacking needed, and if so, how much overlap was there between scans? Also, it would be nice to see what the original slices looked like in the supplement.

Page 11

>"Non-bifurcating branches were found exclusively in the genus Camponotus."

What is a branch that doesn't bifurcate? In the context of this paper, I thought that branching meant bifurcation (or trifurcation, etc.).

Page 12

>"In each case we excluded outliers that were clearly outside the size range of most of our data"

Given that branching is relative, I don't understand why some data were removed.

Page 13

>"we derived an analytical expression for the complete system (each spiracle),"

What does 'each spiracle' mean?

>"Finally, we assumed a CO2 partial pressure difference between the deepest branch level (i.e. level 5) and the external environment to be 6 kPa [22,35]."

Lighton gives a range of 4 to 6 kPa. What happens if the lower limit is considered?

Page 14

>Delete the space between '66 %'.

>"The local partial pressures throughout the airway was evaluated"

Change to 'were'.

>"for ants undertaking different activities (resting walking or running workers) were used."

What does 'resting walking' mean?

>Change 'Tissue level CO2' to 'Tissue-level CO2'.

Page 22

>Which network transport models?

>"By contrast, Nunome [26] acknowledged that insect tracheae may be area-reducing (Nunome's Pattern)."

'Acknowledged' makes it seem as if it were already known. 'suggested'? 'theorized'?

>From the 3D reconstructions and raw slices, I'm struck by how few tracheal tubes are present. I'm not sure what to do with this comment, it just makes me wonder if there was something in the technique that resulted in a collapse of some part of the system. It doesn't conform with what we saw in ants or other insects (beetles or grasshoppers). If you'd like to take a look, here is the full (and short) list of 3D tracheae that we've published:

Pendar, H., J. Aviles, K. Adjerid, C. Schoenewald and J. J. Socha (2019). "Functional compartmentalization in the hemocoel of insects." Scientific Reports 9(1): 6075.

Socha, J. J. and F. De Carlo (2008). Use of synchrotron tomography to image naturalistic anatomy in insects. Developments in X-Ray Tomography VI: 2008, San Diego, CA, SPIE, 70780A-70787.

Socha, J. J., T. D. Förster and K. J. Greenlee (2010). "Issues of convection in insect respiration: Insights from synchrotron X-ray imaging and beyond." Respiratory Physiology & Neurobiology 173(Supplement 1): S65-S73.

I’m also happy to share other images if you’d like to see anything.

Page 25

>Change both instances of ‘whole organism(al)’ to ‘whole-organism(al)’.

Supplement 1

There are many editorial errors in this document. In addition to reading this over carefully, make sure to: change the μ to μm: clear up what “It is obvious that in the cited cases and especially for Tenebrio the numerical results can claim only a very limited accuracy” means; tell us where the details are published; show us figure 2; and use decimals instead of commas in numbers. Also, what makes 7% insignificant?

S2

What data are being referred to? Make sure to include in the legend.

S3

What is this? Include some explanation. Also, formatting errors.

S4

Cite Kestler where appropriate.

S6

Include units where appropriate.

S8

Major and minor what? Plus formatting errors.

S9

Italicize species names. Plus formatting errors.
