# Supplementary material for: Tracheal branching in ants is area-decreasing, violating a central assumption of network transport models
Source: PLoS Comput Biol. 2020 Apr 30;16(4):e1007853. doi: 10.1371/journal.pcbi.1007853 (PMC7241831; doi:10.1371/journal.pcbi.1007853)
Supplement: Attachment — Submitted filename: Aitkenhead_et_al_response.docx [file pcbi.1007853.s012.docx]

Dr Stefano Allesina, Deputy Editor

Dr Van M Savage, Guest Editor

*PLoS Computational Biology*

15^th^ February 2020

Dear Dr Allesina and Dr Savage

**RE: PCOMPBIOL-D-19-01679: Aitkenhead et al.**

Thank you for your e-mail of 17^th^ December 2019, your comments, and those of the two reviewers – Dr Socha and Dr Glazier. We appreciate the enthusiasm for our work and the helpful comments which have helped improve our presentation and its contextualization. We are especially pleased to have had such careful consideration of our work.

We have revised our manuscript and its supplementary sections accordingly. Below we indicate how we have done so in response to the comments raised.

We trust that the work is now suitable for *PLoS Computational Biology*, but also stand ready to discuss any other matters which might arise.

Yours sincerely

**STEVEN L CHOWN**

**On behalf of the authors**

**Responses**

**Guest editor**

The reviewers both appreciate the contributions of this paper and recognize the incredible value of the empirical data and the amount of work it took to obtain these measurements. The connection to allometric theory is also viewed as important. Indeed, the common theme among the reviewers is that there should be more discussion of the details and debates about metabolic theory--though for somewhat different reasons and points. I think the general advice is good. Test acknowledging limitations of allometric theory, that debates exists, the variation exists and possible sources of this variation, and how this all connects to the data in the present paper would be welcome. However, I think this can be kept fairly brief via modifications to existing text and perhaps a few additional paragraphs throughout. I don't think the main themes of the paper needs to change, and I think leading with the main results and dominant views of metabolic theory is appropriate. All other comments by the referees should be addressed and changes made to the paper where appropriate, including correction of typographical errors.

**Response:** We appreciate this guidance and have proceeded in this way. In consequence, we have revised our opening paragraph to consider the literature more broadly, including several of the works to which Dr Glazier refers. We would like to make it clear in this opening response, however, that we do not think this manuscript the place to revisit all of the many arguments concerning which mechanistic approach best explains the scaling of metabolic rate with size, given the empirical data, theoretical assumptions, and diversity of evidence for and against supply versus demand approaches and various others. We really do appreciate this diversity of views and have now made this diversity clear in the opening sections. We also don’t think that we would be well served by revisiting the large variety of mechanistic approaches using our new information. That would be a substantial task and one very different to what we are doing in this paper. Rather, we set out to examine a very specific empirical assumption of the metabolic theory of ecology (as it is now understood to be the approach developed by West, Brown and colleagues). In our view, this is different to metabolic ecology and we hold this view based on Brown et al.’s (2004) *Ecology* paper and the review by one of us (MRK) on metabolic ecology published in *Comprehensive Physiology*. Nonetheless, we do appreciate that we need to recognize the variety of views and have now done so in keeping with the suggestions made by Dr Savage.

**Reviewer 1 (Dr Glazier)**

The authors show that the tracheal transport networks in 20 species of ants are area decreasing, rather than area preserving or slightly increasing, as assumed by resource-transport-network models at the core of the influential metabolic theory of ecology (MTE). Technically, this is a remarkable piece of work. The authors’ findings and their implications for O2 uptake and CO2 release are both useful and noteworthy because they apply to the most diverse major taxon of life, the insects.

**Response:** We appreciate these positive comments and the very helpful further review provided.

However, I have two major concerns, which I believe that the authors should consider.

1) It would be helpful if the authors explicitly addressed the question of whether and how the geometry and physics of transport networks affect the body-mass scaling of metabolic rate. As the authors know, this is a controversial topic, but in my opinion, they do not give sufficient context so that readers can fully appreciate the many dimensions of this controversy and the relevance of their own findings. I am not suggesting a long discussion about this.

However, it would be helpful if readers were made aware that:

(a) Closed resource-transport networks, assumed by the MTE, do not occur in all organisms. Many groups of animals and plants do not have closed resource-transport networks, and some do not have any anatomical transport networks at all. Therefore the MTE, as originally proposed, has limited applicability.

**Response:** We now raise this point in the second paragraph of the Introduction.

(b) Insects have air sacs in their tracheal transport networks, which departs from the network geometry specified by resource-transport-network models at the core of the MTE.

**Response:** We now say so in paragraph four of the Discussion.

(c) Resource-transport-network models of metabolic scaling currently have no direct mechanistic empirical support, unlike some other models based on surface-area exchange of resources/wastes, and size-related changes in cell size/number, various resource-demanding processes, and the relative amounts of tissues with varying metabolic intensities (reviewed in Glazier 2014, 2018a).

**Response:** We have now mentioned contrary findings. We have not gone into them in great detail because we think the works we now cite (including some of those mentioned by Dr Glazier) do the best job of this and we don’t think that this manuscript is the place to revisit these arguments.

(d) Several lines of evidence contradict predictions of resource-transport-network models of metabolic scaling, or are not explained by these models (see e.g., Chown et al. 2007; Glazier 2014, 2015, 2018a; Hirst et al. 2014; Glazier et al. 2015; Harrison 2017).

**Response:** We now allude to this explicitly too, and have cited several of the works mentioned.

2) It would be helpful if the authors considered whether the area-decreasing branching geometry of tracheal transport networks in ants helps to explain their metabolic scaling, if at all. In particular, how do the authors’ findings help explain the diverse metabolic scaling relationships shown by various ant species, as reported previously by one of the authors and his colleagues (Chown et al. 2007). Can this variation in metabolic scaling (slopes ranging between 0.56 and 1.28) be explained by parallel variation in the geometry of their transport networks? If not, the claim that resource-transport-network models of metabolic scaling have no direct mechanistic empirical support still stands. No mention is made of the claim by Chown et al. (2007) that the cell-size model better explains the metabolic scaling of ants than do resource-transport-network models.

**Response:** Without having data on both metabolic rates of the specific species we examined and on the form of the tracheal branching patterns for these species, we don’t think we can do this in a quantitative way. We don’t think we would be well served by a superficial qualitative attempt at doing so either. Indeed, we don’t think it’s worth speculating too broadly given the range of compering explanations which exist for metabolic scaling. To do a proper job of what the reviewer is suggesting would require a large additional study, which was not the point of this empirical work. Nonetheless, we take the point that omitting reference to our previous work (Chown et al. 2007) will seem incongruous to anyone who knows the field (or who decides to explore it). Therefore we do raise the matter now in the final paragraph of the Discussion.

In the last sentence of their summary the authors state: “Our work suggests that much still needs to be done to understand the fundamental assumptions underlying network transport models and how they apply more generally across life – especially in the context of virtually ubiquitous metabolic scaling.” However, nowhere in the main text do the authors discuss the relevance of their findings for our understanding of metabolic scaling in an explicit way.

**Response:** We have now added an extra paragraph at the conclusion of the Discussion which does so.

Specific comments:

Author summary, L 4, 12: Please clarify what is meant by “inwards direction”. Do you mean from the external spiracles towards the inner body core?

**Response:** We do so, explaining that the direction is from the largest to smallest tubes.

Author summary, L 14-15: What is meant by “virtually ubiquitous metabolic scaling”, Are you referring to the 3/4-power law, or merely to the fact that metabolic rate scales with body mass regardless of the slope. Metabolic scaling may be log-log linear or curvilinear with slopes varying from near 0 to > 1. Whatever way the authors view this, how do they think that the geometry of transport networks affects metabolic scaling relationships, if at all? Evidence is growing that transport networks may not exert supply limits on resting metabolic rate, as assumed by the MTE (see e.g., Glazier 2015; Harrison 2017). Some investigators are now arguing that effects of resource demand should be considered in addition to or instead of resource-supply limits (e.g., Glazier 2014, 2018b; Harrison 2017). The question boils down to whether the geometry of transport networks are primary causes or secondary responses to metabolic scaling.

**Response:** We appreciate the diversity of scaling exponents, views on the mechanisms underlying the scaling of metabolic rate with body size, and diversity of opinion on whether or not central tendencies exist and how important variation about them is. We do not intend to generate a further debate in the author summary and have simply now changed our text to state the following:

*Our work suggests that much still needs to be done to understand the fundamental assumptions underlying network transport models and how they apply more generally across life –* *especially in the context of why metabolic rate scales with body size.*

Methods (p 12): In OLS regression analyses, it is assumed that there is a dependent (Y) and independent variable (X). Is ri or rj the X variable, and if so what is the justification?

**Response:** We don’t think that this matters particularly, but in doing so are following previous convention (see for example the work by Price et al. 2013 *PLoS One*, which we cite (now as ref 21)).

Fig. 2b, e: Please make sure that the features designated in these pictures are more easily visible to readers.

**Response:** We have adapted figure 2 to include larger examples of the raw slices, as well as enhancing the contrast to ensure the features are more easily visible to readers.

Fig. 3a: Are the OLS “outcomes” slopes? If so, please say so. Also please make clear what the X and Y variables are for these regressions. From reading the text (e.g. on P 4), I assume that the variables are ri and rj (radii of the parent and child vessels), but I am not sure which was considered X versus Y. I am having difficulty matching up the data shown in panel ‘a’ with those shown in panel ‘b’.

**Response:** These are the slopes, which is now indicated in the text. The sum of the area of child branches was used as the dependent variable (Y) and the area of the parent branch was used as the predictor (X). This is now indicated in the text and in the figure caption. We have looked over Figure 3 repeatedly, discussed it with colleagues and among ourselves, and have not come to a conclusion about what the final sentence of this comment means. We have thus not made any further changes, but would be pleased to take editorial direction on this matter if we have somehow missed the point.

References:

Chown, S. L., Marais, E., Terblanche, J. S., Klok, C. J., Lighton, J. R. B., & Blackburn, T. M. (2007). Scaling of insect metabolic rate is inconsistent with the nutrient supply network model. Functional Ecology, 21(2), 282-290.

Glazier, D. (2015). Body-mass scaling of metabolic rate: what are the relative roles of cellular versus systemic effects? Biology, 4(1), 187-199.

Glazier, D. (2018a). Rediscovering and reviving old observations and explanations of metabolic scaling in living systems. Systems, 6(1), 4.

Glazier, D. S. (2018b). Resource supply and demand both affect metabolic scaling: A response to Harrison. Trends in Ecology & Evolution, 33(4), 237-238.

Glazier, D. S., Hirst, A. G., & Atkinson, D. (2015). Shape shifting predicts ontogenetic changes in metabolic scaling in diverse aquatic invertebrates. Proceedings of the Royal Society B: Biological Sciences, 282(1802), 20142302.

Harrison, J. F. (2017). Do performance–safety tradeoffs cause hypometric metabolic scaling in animals?. Trends in Ecology & Evolution, 32(9), 653-664.

Hirst, A. G., Glazier, D. S., & Atkinson, D. (2014). Body shape shifting during growth permits tests that distinguish between competing geometric theories of metabolic scaling. Ecology Letters, 17(10), 1274-1281.

**Reviewer 2 (Dr Socha)**

This manuscript is a truly exciting piece of work. Ever since the first high-profile papers of the (poorly named) metabolic theory of ecology came out, I have scratched my head over the claims that insect tracheal systems conform to their hypothesis. There were so little data on tracheal branching patterns in insects (the compilation in S6 documents this well; it’s great to see), and so many species, and so much variation, that really insects should have been considered as an unknown. The current work examines branching patterns in 20 species of insects and finds a surprising result: that cross-sectional area decreases down the network. Additional modeling adds another surprise: that CO2 is really the culprit, not O2. Just fascinating. And I would note that while collecting synchrotron tomography data is relatively fast (compared to classical techniques), the data analysis is not fast at all. The data and analysis presented must have taken a very large eﬀort, and I hope that that is not lost on readers. Overall, I think that this is a very important manuscript, and it should have a large impact in the literature.

**Response:** We appreciate the positive response and in particular the recognition that it has taken us several years to process the synchrotron data we collected.

Despite my great enthusiasm, I have many specific comments for improvement of the manuscript, documented below. And I have two general comments:

1) The language about what the exponents ‘should’ be is not quite right. There is not just one set of values that provide area constant or area increasing characteristics; other values are possible. (In fact we find intermediate values in the beetle we studied, in a manuscript that is soon to be submitted.)

**Response:** We don’t doubt that the values need not be exact and that there’s variation to be expected, which may arise either from measurement error, or because of the way the system has evolved in a given species. Nonetheless, it is pretty clear that there are limits and these limits have particular meanings. We have not seen the manuscript concerned which shows intermediate values. Nonetheless, to accommodate the reviewer’s view, we have adjusted our language in the final paragraph of the Introduction to reflect this.

2) The supplements didn’t seem to be drafted with the same level of care as the rest of the manuscript, and need improvement. (Details below.)

**Response:** We have reformatted all of the Supplementary Information, transformed all of these sections into PDFs and made sure that the formatting matters raised throughout have all been addressed. Initially the conversions from Excel Tables to PDF did not work very well, but we have redone all of those tables in Word and that has solved the problem.

Page 2:

>“The assumption of such a pattern underpins one of biology’s most influential ideas – the metabolic theory of ecology.”

Add a citation for the latter part of the sentence.

**Response:** Our understanding is that the author summary does not include citations. If this is not the case then we would be more than happy to provide a reference.

Page 3:

“with a transition to an increase in total cross-sectional area of smaller vessels (also known as Murray’s law) [1,8]”

>“Thus, the summed cross-sectional area of the child branches is either the same as or larger than the cross-sectional area of the parent branch (Fig 1).”

Given that the previous sentence discussed several options, using “thus” does not make sense here.

**Response:** We have removed the word.

Page 4:

>“What the situation is for insects remains poorly known, resting, in discussions of metabolic scaling, on early empirical work [12] suggesting that branching is area-preserving (based on a single individual insect; Supporting Information S1), and on an assumption that diﬀusion dominates gas exchange through the tracheal system [1,13], which is the main transport route for O2 to insect tissues and for CO2 eﬄux from the body [14].”

This long sentence is a bit disappointing given the clear, elegant argument that was made in the cover letter. I would suggest expanding this a bit and borrowing simpler language from the letter, which is far more compelling.

**Response:** We have now re-written this section as requested.

>“Specifically, we tested the prediction that, in the equation relating parent to child branches:

𝑟 𝑟 𝑟 (1)

α should be 2 or 3, as is the case for area-preserving and area-increasing branching patterns,”

There’s something oﬀ about the language here, but I can’t quite articulate what it is. There is no reason why alpha should be 2 or 3 in insect tracheal systems, unless there is a specific hypothesis of function. The condition where cost of flow and investment in wall material are both minimized, alpha is 3, but that does not mean that alpha should be 3; if it were found, it would suggest that evolution drove branching patterns in that direction.

**Response:** We do not quite understand the concern here. We agree with the argument and the rationale for these assumptions as set out, for example, in the Price et al. work we cite (now Ref. 21). We are dealing with specific hypotheses of function set out by the MTE and by the arguments originally made for Murray’s Law. Perhaps we have somehow misunderstood this comment. We have altered our text to clarify.

Page 6

>“Using tracheal network data from our investigations, along with partial pressure variation data from the literature [20,22], the modelling indicates that tracheal architecture with an inwards decline in cross-sectional area with branching (which means area-increasing branching outwards), delivers the highest potential CO2 flux, in keeping with empirical data on ant metabolic rates (Fig 3c; Supporting Information S5).”

This sentence could be split up to help improve clarity.

**Response:** We have re-written this section as requested.

>“Thus, for the 30 species of insects that have been examined for cross-sectional area variation among…”

This ‘thus’ doesn’t fit here, given the previous sentence.

**Response:** We removed the word.

Page 7

>“Hereafter we term a pattern of area reduction with branching ‘Nunome’s pattern’, acknowledging his early recognition thereof [26].”

I was thrown oﬀ here, because I felt like I missed something earlier about Nunome. This is the first mention of his name, so perhaps the explanation should come before the naming of the pattern.

**Response:** We see the point and have moved this naming of the pattern to the third paragraph of the Results, where we now see it is more effective.

>“In consequence, such a transport network should provide a biophysical advantage proximally, and ultimately a fitness benefit.”

I think that ‘physiological’ is more appropriate than ‘biophysical’; the former encompasses the latter.

**Response:** We have altered the word as required.

>“In consequence tracheal systems are likely to be optimised for CO2 transport outwards.”

In the context of this paragraph, ‘specialized’ might be more appropriate than ‘optimised’, which is diﬃcult to prove in an evolutionary sense. Also, add a comma after ‘consequence’.

**Response:** We don’t doubt the difficulty of the proof, but that’s no reason not to suppose evolution has been involved. Optimized is what we mean and, with respect, have retained the word.

>“accounting for the empirical data demonstrating that Murray’s law is rare in insects (Supporting Information S6).”

S6 seems to indicate that Murray’s law has not been found in insects. Our study (cited as an abstract) is about to be submitted for review and is might be better cited here as in review, if allowed. If not, Kenny’s 2019 PhD thesis (“Structural and physical characterization of insect flow systems”) is a better reference.

**Response:** We are a little reluctant to change the citation to a PhD thesis or to a submitted ms. We understand the reviewer’s preference for this, but at the same time we think that for the moment the published, and/or reviewed, accessible sources are best. If the reviewer is suggesting that the work we have not seen supports Murray’s law, then that changes matters, but Dr Socha does not say so.

Page 8

>“Our data for the more proximal tracheal branches also show that such branching patterns are not simply restricted to more distal elements of the system as suggested recently [27].”

I don’t seem to remember a statement to that eﬀect in that paper, but I could be wrong.

**Response:** Jon Harrison, in reviewing a draft of this work, pointed us to his 2018 work published in the *Journal of Insect Physiology*. We are basing our statement here on the Discussion section of this paper and it’s Figure 8, specifically p. 195, right-hand column, fourth paragraph: *It has been shown in some insects that the axial diffusing capacity or summed cross-sectional area of successive generations is constant, allowing development of simple models of gas diffusion for insects (Krogh, 1920; Locke, 1958; Thorpe and Crisp, 1947). In contrast, we found that, for the tracheae next to and within the flight muscle, the axial diffusing capacity decreases strongly with each successive generation (Fig. 8). The cited prior studies that found constant summed cross-sectional areas across generations focused on larger tracheae.*

>Regarding the capitalization of ‘x-ray’: Although there is some debate about style, “x-ray” is probably better depicted without the capital letter. This is a minor matter, but see here for debate: https://english.stackexchange.com/questions/9891/should-the-x-in-x-ray-be- capitalized/9899#9899

**Response:** We have changed to ‘x-ray’ throughout.

>“Irrespective, two major empirical assumptions of transport network models, that branching is either area-preserving…”

There are loads of network transport models in the biological and engineering literature. Be specific here.

**Response:** Changed to the MTE.

>“They were then exposed to iodine vapour for 7 days to improve contrast for imaging [37], which resulted in death.”

I’m very interested in this technique and will try it myself, but I wonder why it was needed in the first place. Generally with x-ray tomography (both synchrotron and tabletop), the contrast between air and tissue is quite high, and there’s no real need to do anything to the tissue. If you look at the first synchrotron tomography images of insect tracheal systems made in the mid 2000’s, the contrast was quite high. Overall, I’m somewhat surprised by the slice images shown in figure 2, but they are good enough for the analysis.

For reference, the referred images are in the following paper (which is obscure but can be downloaded from my lab’s website): Socha, J. J. and F. De Carlo (2008). Use of synchrotron tomography to image naturalistic anatomy in insects. Developments in X-Ray Tomography VI: 2008, San Diego, CA, SPIE, 70780A-70787.

**Response:** We agree: the contrast between air and tissue is generally sufficient enough by itself to be able to differentiate the tracheal system without the need for staining.

Staining was done under the advice of the Australian Synchrotron staff. These were the first insect samples to be scanned on their lines, and staff advised that staining may help the detector “Ruby” achieves its best possible resolution. Of the staining methods we had tested on benchtop sources (phosphotunstic acid, aqueous iodine), the method using vapour proved the best and to have no impact on the airspaces within the animal as the other methods did.

>“in preparation for Synchrotron X-Ray tomography” No capitals needed.

**Response:** Changed as requested.

Page 10

>More details needed. What was the exposure time per image, and how long did a scan take to collect? This duration has an impact on ability to control motion in the tracheal system (induced by heating or other things). How many scans per insect? What was the field of view for the raw projections images? Was vertical stacking needed, and if so, how much overlap was there between scans? Also, it would be nice to see what the original slices looked like in the supplement.

**Response:** We have expanded our methods section to include these details. Here in brief, exposure time was 0.033 seconds with 1800 images taken during a single scan of each individual. Vertical stacking was needed due to the mounting method with the overlap being 110 pixels. Because of this the total time an individual was exposed to the beamline was 6 minutes (3 minutes per vertical section).

We have also included a further supplement (Supporting Information S10) with an original slice from a representative individual of each genus studied (the only manipulations being to convert to 8 bit rather than 32 bit to save on file size for submission, and to enhance the contrast to ensure readers can see the different structures).

Page 11

>”Non-bifurcating branches were found exclusively in the genus Camponotus.”

What is a branch that doesn’t bifurcate? In the context of this paper, I thought that branching meant bifurcation (or trifurcation, etc.).

**Response:** We have rephrased this to be more clear.

Page 12

>”In each case we excluded outliers that were clearly outside the size range of most of our data”

Given that branching is relative, I don’t understand why some data were removed.

**Response:** We wished to be as conservative as possible with the regression approaches by not introducing points which could have marked leverage on the outcomes, which may occur when points are far outside the typical data range, and which we thought might be in error for some reason.

Page 13

>”we derived an analytical expression for the complete system (each spiracle),” What does ‘each spiracle’ mean?

**Response:** We have now pointed out that this is the tracheal system inwards from each spiracle.

>”Finally, we assumed a CO2 partial pressure diﬀerence between the deepest branch level (i.e. level 5) and the external environment to be 6 kPa [22,35].”

Lighton gives a range of 4 to 6 kPa. What happens if the lower limit is considered?

**Response:** We think the reviewer missed our exploration for outcomes of 4 kPa and 8 kPa that were originally presented in Supplementary Information S5 and mentioned in the Methods Section. We have retained these explorations. To prevent this from happening to other readers, we have now also included a reference to S5 in the legend to Figure 3,

Page 14

>Delete the space between ’66 %’.

**Response:** Done.

>”The local partial pressures throughout the airway was evaluated” Change to ‘were’.

**Response:** Done

>”for ants undertaking diﬀerent activities (resting walking or running workers) were used.” What does ‘resting walking’ mean?

**Response:** We have inserted a comma between resting and walking, which clarifies the matter.

>Change ‘Tissue level CO2’ to ‘Tissue-level CO2’. Page 22

**Response:** Done

>Which network transport models?

**Response:** We now say the MTE.

>”By contrast, Nunome [26] acknowledged that insect tracheae may be area-reducing (Nunome’s Pattern).”

‘Acknowledged’ makes it seem as if it were already known. ‘suggested’? ‘theorized’?

**Response:** We have changed the word to ‘proposed’.

>From the 3D reconstructions and raw slices, I’m struck by how few tracheal tubes are present. I’m not sure what to do with this comment, it just makes me wonder if there was something in the technique that resulted in a collapse of some part of the system. It doesn’t conform with what we saw in ants or other insects (beetles or grasshoppers). If you’d like to take a look, here is the full (and short) list of 3D tracheae that we’ve published:

Pendar, H., J. Aviles, K. Adjerid, C. Schoenewald and J. J. Socha (2019). "Functional compartmentalization in the hemocoel of insects." Scientific Reports 9(1): 6075.

Socha, J. J. and F. De Carlo (2008). Use of synchrotron tomography to image naturalistic anatomy in insects. Developments in X-Ray Tomography VI: 2008, San Diego, CA, SPIE, 70780A-70787.

Socha, J. J., T. D. Förster and K. J. Greenlee (2010). "Issues of convection in insect respiration: Insights from synchrotron X-ray imaging and beyond." Respiratory Physiology & Neurobiology 173(Supplement 1): S65-S73.

I’m also happy to share other images if you’d like to see anything.

**Response:** We agree that there are relatively few trachea in the 3D renders, the imagery shown is of the tracheal system modelled to the 3^rd^ level, this is because this was the lowest level measured in this study. Examples from the literature may go as far down as the 6^th^ level (from our count), so one expects to see at least double the number of trachea in those images as in ours.

To account for this to some extent we have adapted figure 2 to include better examples of raw slices with more trachea present to prevent readers from thinking this may be the case. We have also included a new supplement S10 which allows readers to see a raw scan slice of a representative individual for each genus studied, and so the amount of tracheation present in an individual is also more clearly illustrated.

Page 25

>Change both instances of ‘whole organism(al)’ to ‘whole-organism(al)’.

**Response:** We have made the changes.

Supplement 1

There are many editorial errors in this document. In addition to reading this over carefully, make sure to: change the µ to µm: clear up what “It is obvious that in the cited cases and especially for Tenebrio the numerical results can claim only a very limited accuracy” means; tell us where the details are published; show us figure 2; and use decimals instead of commas in numbers. Also, what makes 7% insignificant?

**Response:** We appreciate that there may be errors of various kinds in the opinion of Dr Socha, but we would like to politely remind him that we are providing here a translation to English, in as accurate a form possible, of what Krogh wrote in German. We have not sought to alter the text, style and grammar (more than we think reasonable from the older German), and have not sought to try to interpret Krogh’s thinking. We have now added an explanation to this effect and, to make sure that the interpretation is clear, we have reproduced below the English the exact German text extracted from Krogh’s work. We think it would cast doubt on the veracity of our translation if we amended the units or Krogh’s lack of use of italics and so on.

S2 What data are being referred to? Make sure to include in the legend.

**Response:** We have now added additional information to the title to make sure that the data source is clear. We also make clear which level is considered X and which Y in the regressions. Note the Major Axis approach is provided because of measurement error in both as we explain in the Methods.

S3 What is this? Include some explanation. Also, formatting errors.

**Response:** We have now included a full explanation and have reformatted the information.

S4 Cite Kestler where appropriate.

**Response:** We have done so.

S6 Include units where appropriate.

**Response:** We have done so. We note that Krogh did not supply units, and that ratios have no units. In the original we included units in cm^2^ at the end of each section and that may have been confusing. We have corrected this matter

S8 Major and minor what? Plus formatting errors.

**Response:** We explain these are minor and major workers and have corrected the formatting errors.

S9 Italicize species names. Plus formatting errors.

**Response:** We have corrected all errors and formatted species names in italic font.
