# Supplementary material for: Tracheal branching in ants is area-decreasing, violating a central assumption of network transport models
Source: PLoS Comput Biol. 2020 Apr 30;16(4):e1007853. doi: 10.1371/journal.pcbi.1007853 (PMC7241831; doi:10.1371/journal.pcbi.1007853)
Supplement: Attachment — Submitted filename: Aitkenhead_et_al_response_2.docx [file pcbi.1007853.s013.docx]

 Dr Stefano Allesina, Deputy Editor

Dr Van M Savage, Guest Editor

*PLoS Computational Biology*

1^st^ April 2020

Dear Dr Allesina and Dr Savage

**RE: PCOMPBIOL-D-19-01679R1: Aitkenhead et al.**

Thank you for your e-mail of 1 April 2020, your comments, and those of the two reviewers – Dr Socha and Dr Glazier. We appreciate the second round of review and that during this coronavirus pandemic some delay to reviews was inevitable. We are grateful for the work done by the reviewers and especially by Dr Socha since we were in direct touch with him about his abrupt change in arrangements. Almost everyone teaching and/or looking after animals is in a similar position.

We have revised our manuscript and Supporting Information S1 as requested. Below we indicate how we have done so in response to the comments raised.

We trust that the work is now suitable for *PLoS Computational Biology*.

Yours sincerely

**STEVEN L CHOWN**

**On behalf of the authors**

**Responses**

**Editor**

We have made all the changes required and re-checked our ms for compliance with the formatting instructions. Both a marked-up and clean version of the revision have been submitted. Most of the supporting information and figure files did not need to be changed so we have retained those from the previous versions. The figures had already been checked via the PACE system.

**Reviewer 1**

Reviewer #1: Great piece of work! The authors have effectively dealt with all of my concerns. I look forward to the day when direct comparisons between the geometry of resource-transport networks and the scaling of metabolic rate are made across individual species, a huge but necessary task for evaluating the validity of resource-transport network models of metabolic scaling.

**Response:** Thank you for the comprehensive initial review which has provided us with an improved manuscript.

**Reviewer 2**

Reviewer #2: I apologize to the authors for my delay. The Covid-19 crisis hit the US in the past few weeks, and as with many of us, I scrambled to rework my classes and deal with other things as a consequence.

The authors have made a great number of changes to improve the manuscript, and I have no further major comments. I do have a few minor suggestions below. I stand by my original assessment that this manuscript is an exciting piece of work, and I applaud the authors for their efforts in going above and beyond to provide a great amount of detail, useful scholarship for the field. Congratulations.

**Response:** Thanks for the work on this, especially given the many other unexpected demands on your time given changing circumstances to do with the pandemic. We appreciate the very helpful and thoughtful comments during both rounds of review.

“Interpretation of the tracheal system as one optimized for the release of carbon dioxide, while readily catering to oxygen demand, explains the branching pattern.”

In the abstract here, having not read the study, it’s not clear if mechanical optimization or evolutionary optimization is intended. As these mean different things and are analyzed differently, it would be helpful to indicate that here. (I assume mechanical is meant.)

**Response:** Thank you for this comment. We had actually given this some thought previously. In a strictly human-manufactured system, writing ‘mechanical optimization’ would be reasonable. For animals, however, any optimization must involve evolution at some point along the trajectory of change in tracheal systems. In consequence, not being specific here is in our view the best course of action since, although we have not conducted experiments that have shown laboratory evolution, the implication from what we do show is that evolution must have been involved at some point. Thus, we have not made any changes.

Our results, together with widespread demonstration that gas exchange in insects includes, and is often dominated by, convection, indicate that for generality, network transport models must include consideration of systems with bidirectional flow.”

The directionality doesn’t matter, per se. The same argument could apply for grasshoppers, which partially use unidirectional flow.

**Response:** We have modified the sentence to: ‘Our results, together with widespread demonstration that gas exchange in insects includes, and is often dominated by, convection, indicate that for generality, network transport models must include consideration of systems with different architectures.

“models do not apply in all circumstances, and often concluding that other approaches have”

Change ‘concluding’ to ‘conclude’.

**Response:** Changed.

”In consequence, tracheal systems are likely to be optimised for CO2 transport outwards.”

Same comment about mechanical optimization applies here.

**Response:** Please see our response above where we dealt with this question.

”Until the implications … is explored further,”

Change to ‘are explored’.

**Response:** Changed.

Thank you for supplying details of the imaging and providing sample slices in the supplement; it is all useful to see. One more question: what is the nominal resolution of the system? The value of 0.15 µm difference described below should be at or below the possible resolution.

**Response:** Good point. This is a statistical difference not an actual one. Thus, we now write: ‘Measurements were validated by remeasuring the full dataset and comparing first and second measurement values: across the full data set of 1258 measurements, no measures differed statistically by more than 0.15 µm (below the nominal resolution of the system of 7.6 µm), with a mean difference of 0.05 µm. The original measurements were used for analysis.’

Also, as there are very few studies of tracheal systems using synchrotron tomography, I think it would be useful to point to the first one (Socha and De Carlo 2008), because the methods of preparation described here are different (and were also used in Harrison et al 2018). Of course no strict need to do this; I don’t mean to insist that you cite my paper, by any means.

**Response:** We now cite this work, thanks.

”A simple model of steady state diffusive gas exchange”

Change to ‘steady-state’.

**Response:** Done.

Supplement 1

I apologize for my original comments about editorial errors in the document. Somehow when I was reading it I missed the header, indicating that it was a translation. Clearly I was reading too quickly, and I thought that the text described a comparison to his original study. I actually think it’s a great service to the field to provide the translation in this paper, so I thank you. (Many years ago, I believe that I sat down with a German speaker to discuss the paper, but I didn’t have a written translation to work with.) For completeness, it would be nice to provide the name of the person who translated it as credit at the top, as well as the full original citation and translated title.

**Response:** We have done so.

Supplement 6

Just a side note in response to the comment, “We note that Krogh did not supply units, and that ratios have no units.” Ratios are not necessarily dimensionless; for example, speed is a ratio that has units of length per time.

**Response:** Thanks, we will keep this in mind.
